# PIK3C2A is a prognostic biomarker that is linked to immune infiltrates in kidney renal clear cell carcinoma

**DOI:** 10.3389/fimmu.2023.1114572

**Published:** 2023-03-30

**Authors:** Chengdong Qin, Siyuan Liu, Shiqi Zhou, Qibo Wang, Xianghou Xia, Jiejie Hu, Xiaohong Yuan, Zongping Wang, Yang Yu, Dening Ma

**Affiliations:** ^1^ Department of Breast Surgery, Zhejiang Cancer Hospital, Institute of Basic Medicine and Cancer (IBMC), Chinese Academy of Sciences, Hangzhou, Zhejiang, China; ^2^ Department of Colorectal Surgery, Zhejiang Cancer Hospital, Institute of Basic Medicine and Cancer (IBMC), Chinese Academy of Sciences, Hangzhou, Zhejiang, China; ^3^ Department of Urology Surgery, Zhejiang Cancer Hospital, Institute of Basic Medicine and Cancer (IBMC), Chinese Academy of Sciences, Hangzhou, Zhejiang, China; ^4^ Department of Anesthesiology, Zhejiang Cancer Hospital, Institute of Basic Medicine and Cancer (IBMC), Chinese Academy of Sciences, Hangzhou, Zhejiang, China; ^5^ Key Laboratory of Prevention, Diagnosis and Therapy of Upper Gastrointestinal Cancer of Zhejiang Province, Hangzhou, China

**Keywords:** PIK3C2A, immunization, prognosis, biomarker, kidney renal clear cell carcinoma

## Abstract

**Background:**

Phosphoinositide 3-kinases (PI3Ks) are lipid enzymes that regulate a wide range of intracellular functions. In contrast to Class I and Class III PI3K, which have more detailed descriptions, Class II PI3K has only recently become the focus of functional research. PIK3C2A is a classical member of the PI3Ks class II. However, the role of PIK3C2A in cancer prognosis and progression remains unknown.

**Methods:**

The expression pattern and prognostic significance of PIK3C2A in human malignancies were investigated using multiple datasets and scRNA-seq data. The PIK3C2A expression in renal clear cell carcinoma (KIRC) was then validated utilizing Western blot. The functional role of PIK3C2A in KIRC was assessed using combined function loss experiments with *in vitro* experiments. Furthermore, the correlation of PIK3C2A expression with tumor immunity was investigated in KIRC. The TCGA database was employed to investigate PIK3C2A functional networks.

**Results:**

Significant decrease in PIK3C2A expression in KIRC, demonstrated that it potentially influences the prognosis of diverse tumors, particularly KIRC. In addition, PIK3C2A was significantly correlated with the T stage, M stage, pathologic stage, and histologic grade of KIRC. Nomogram models were constructed and used to predict patient survival based on the results of multivariate Cox regression analysis. PIK3C2A knockdown resulted in significantly increased KIRC cell proliferation. Of note, PIK3C2A expression demonstrated a significant correlation with the infiltrating levels of primary immune cells in KIRC.

**Conclusion:**

These findings support the hypothesis that PIK3C2A is a novel biomarker for tumor progression and indicates dynamic shifts in immune infiltration in KIRC. Furthermore, aberrant PIK3C2A expression can influence the biological activity of cancer cells.

## Introduction

Renal cell carcinoma (RCC), which arises from the renal epithelium, accounts for approximately 90% of kidney tumors and 3% of all cancers (1), and the past several decades have seen a significant increase in its mortality rate (2). Renal clear cell carcinoma (KIRC) is the most common pathological subtype of RCC, which accounts for 75–80% of all cases (3). KIRC treatment has been modified over the past few decades owing to emerging research into its biology and pathogenesis. Nonetheless, 25% to 30% of patients are characterized by distant metastases at the time of initial diagnosis, and about 40% experience recurrence following surgical resection (4). In this view, an in-depth understanding of the molecular process holds great promise for KIRC prognosis and treatment.

The immune system protects the body’s homeostasis against exogenous and endogenous, infectious and non-infectious threats, as well as eliminating defective cells before they develop into malignant cells. A subset of malignant cells may evade immune cell surveillance, resulting in tumorigenesis and metastatic spread. Interaction of infiltrating immune cells, one of the major components of the tumor microenvironment, with cancer cells has been shown to influence tumor onset and progression (5, 6). Compelling evidence also indicates that the level of immune cell infiltration in tumor and paracancerous tissues may serve as an indicator to predict prognosis with higher accuracy than the standard clinicopathological parameters (7).

Phosphoinositide 3-kinases (PI3Ks) are a type of enzyme that catalyzes the formation of second messengers for a variety of signaling pathways (8). This family can be classified into three subtypes based on structure and substrate specificity (9). The functions of class II PI3Ks are less well understood than those of class I and class III PI3Ks. As a typical member of PI3K class II, PIK3C2A is an abundantly expressed lipid kinase with critical functions during organism development (10). PIK3C2A has been implicated in a number of cancer pathophysiological pathways due to its ability to catalyze the phosphorylation of lipids phosphatidylinositol (PI) into PI (3)P and PI (4)P into PI (3, 4) P2 (11). However, the roles of PIK3C2A in the progression of cancer remain unknown understood. As such, the present investigation seeks to elucidate the role and mechanism of PIK3C2A in tumor progression.

The prognostic landscape of PIK3C2A was investigated using multiple databases. The correlation of PIK3C2A expression with infiltrating immune cells in various types of cancer was analyzed using the TIMER, TCGA, and GEPIA databases. Utilizing loss-of-function assays, the effect of PIK3C2A on the proliferation of KIRC cells was evaluated *in vitro*. Last but not least, the underlying mechanism of PIK3C2A was evaluated using the TCGA database. The findings of this research shed light on the effects of PIK3C2A on KIRC prognosis.

## Materials and methods

### PIK3C2A expression analysis

RNA sequencing data for 33 cancer types were retrieved from the Genomic Data Commons (GDC) data portal website (https://portal.gdc.cancer.gov/) and analyzed using R v4.0.3. Screening distinct mRNAs was performed with the R package ‘DESeq2’ (12); the threshold value was determined to be log2 fold change>1.0 and an adjusted *P*-value of 0.05 unless otherwise specified. With mRNAseq data from the TIMER database, we compared PIK3C2A expression in cancer tissues versus adjacent tissues (13).

### Single-cell RNA sequencing analysis

The scRNA-seq data were analyzed using R (version 4.0.3) with the Seurat package (version 3.1.1). Filtering was performed on sample KIRC cells with < 200 and > 2500 genes and a mitochondrial gene percentage of >10%. The top 2000 most variable genes in the scRNA-seq data were used for principal components analysis (PCA), and the first 20 principal components were used for UMAP analysis. The FindClusters function divided all cells into 14 clusters, with resolution = 0.5.

### Evaluating the connection between PIK3C2A and the prognosis of various tumors

Univariate Cox regression analysis was used to assess the relationship between PIK3C2A and the prognosis of malignancies. Based on the median expression value of PIK3C3A, KIRC survival curves were generated using the ‘survival’ R package. PIK3C2A was evaluated *via* multivariate Cox regression to determine its independence as a predictor of patient survival. Nomogram-based models for predicting patient survival were developed using the R package ‘rms’. The area under the time-dependent ROC curve (AUC) was generated using the ‘timeROC’ (version: 0.4) package to examine the sensitivity and specificity of PIK3C2A in survival prediction.

### Cell lines

The human KIRC cell lines 769P, CAKI-1, 786-O, and ACHN cells were obtained from the cancer institute of Zhejiang Caner Hospital. KIRC cell lines were grown in RPMI-1640 medium (BasalMedia, China) containing 10% fetal bovine serum (FBS), 100 units of penicillin, and 50 units of streptomycin per ml.

### PIK3C2A siRNA construction and transfection

PIK3C2A siRNA was purchased from Zorinbio Company (China); the PIK3C2A siRNA1 sequence was 5’-CCACUUAUGCUUUACCUUCUATT-3’, the PIK3C2A siRNA2 sequence was 5’-GCUAGUGUGAAGGUCUCCAUUTT-3’, and the PIK3C2A siRNA3 sequence was 5’-GAUGACAGUUUCGAGACUAAATT-3’. Lipofectamine 2000 was used to transfect KIRC cells with siRNA. Control cells were transfected with non-targeting siRNA.

Real-time PCR assay

The TRIzol Reagent (Sigma, St. Louis, MO) was used to isolate total RNA, and the PrimeScript RT Reagent Kit was used to reverse-transcribe this RNA into cDNA, per the manufacturer’s instructions (TaKaRa, Dalian, China). The mRNA expressions of PIK3C2A and GAPDH were measured using an RT-PCR quantitative PCR kit (TaKaRa, Dalian, China).

### Western blotting

Cells were lysed in ice-cold RIPA buffer containing protease and phosphatase inhibitors. Proteins from each group were separated using SDS-PAGE and transferred in equal amounts to polyvinylidene fluoride (PVDF) membranes. The membranes were blocked with 5% fat-free milk for one hour and then treated overnight at 4°C with PIK3C2A Mouse mAb (ZENBIO, China). The membranes were then washed thrice in Tris-buffered saline with Tween-20 (TBST) and incubated at room temperature for 1 hour with a secondary antibody conjugated to HRP. Blots were examined with an ECL detection kit (NCM Biotech, China) and processed with Quantity One 1-D Analysis Software (Bio-Rad, San Francisco, CA, USA).

### Colony formation and cell proliferation assays

Colony formation tests were performed as described previously (14). Briefly, 3000 cells/well were seeded in 96-well plates, and cell proliferation was evaluated using a Cell Counting Kit-8 (CCK-8; NCM Biotech, China), measuring absorbance at 450 nm based on the instructions.

### EdU assay

For cell proliferation assessment, a 5-ethynyl-20-deoxyuridine (EdU) test kit was utilized (Beyotime Biotechnology, China). KIRC cells (2 × 10^4^ cells/well) were seeded in the glass bottom dish and cultured overnight. Afterward, the cells were incubated with 10 μM EdU buffer at 37°C for 2 hours, fixed with 4% formaldehyde for 15 minutes, permeabilized with 0.3% Triton X-100 for 25 minutes, and nuclear-stained with Hoechst. The cells were then examined using a fluorescence microscope. The ratio of EdU-positive cells (red cells) to all Hoechst-positive cells (blue cells) was defined as the EdU incorporation rate.

### Correlation analysis between PIK3C2A and immune infiltration

The TIMER database was utilized to evaluate the degree of relatedness between PIK3C2A and infiltrating immune cells, based on how certain markers show up in different tumors. Heat plots were used to show the correlation between PIK3C2A and each immune gene marker; PIK3C2A was plotted on the x-axis, immune markers on the y-axis, and the gene level was adjusted with log2 TPM. The correlation coefficient between PIK3C2A and immune markers was calculated using the Spearman method in GEPIA.

CIBERSORT is a deconvolution algorithm that can be used to describe the cell composition of complex tissues. Fluorescence-activated cell sorting (FACS) has confirmed that this protocol quantifies a terrific amount of specific cell types (15). Thereupon, CIBERSORT was applied to figure out the relative proportions of 22 infiltrating immune cell types in the PIK3C2A high-expression and low-expression groups, using the LM22 signature matrix and 1000 permutations. Immunedeconv, an R package that contains the CIBERSORT algorithm, was used to carry out this procedure (16).

### Functional and pathway enrichment analysis

The association of PIK3C2A with protein-coding genes (PCGs) was evaluated using Spearman correlation coefficients, and genes with a Spearman correlation coefficient greater than 0.50 were identified as PIK3C2A-associated PCGs. GO and KEGG pathway enrichment studies on the PIK3C2A-associated PCGs were then performed using the clusterProfiler program (17). P-values <0.05 were set as the threshold for GO/KEGG pathway enrichment analyses. The enrichment outcomes were visualized in R using the ggplot2 package.

### Genes set enrichment analysis

The underlying signaling pathways associated with PIK3C2A in KIRC were identified by gene set enrichment analysis (FDR 0.25, adjusted *p*-value 0.05), based on the expression patterns between the high-expression and low-expression groups. This analysis was conducted with the clusterProfile application (17). The PIK3C2A-related pathway in the Molecular Signatures Database (MSigDB) was visualized using the R program ‘ggplot2’.

## Results

### Expression levels and prognostic roles of PIK3C2A in various tumor types

**Figure 1:**
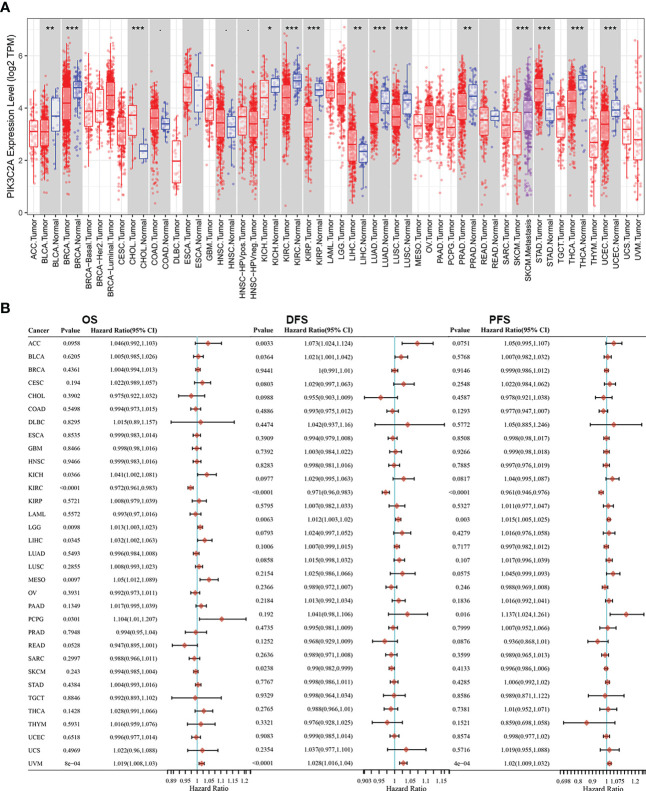
PIK3C2A expression levels and prognostic roles in different types of human cancers. **(A)** Increased or decreased expression of PIK3C2A in different cancer tissues, compared with normal tissues in TIMER. *p < 0.05, **p < 0.01, ***p < 0.001. **(B)** Hazard ratio and p-value of PIK3C2A involved in univariate COX regression analysis and the OS, DFS, and PFS of various malignancies.

The TIMER database was employed to compare the levels of mRNA for PIK3C2A in various kinds of tumors and nearby tissues. Compared with normal controls, PIK3C2A expression was significantly lower in bladder urothelial carcinoma (BLCA), breast invasive carcinoma (BRCA), kidney chromophobe (KICH), kidney renal clear cell carcinoma (KIRC), kidney renal papillary cell carcinoma (KIRP), lung adenocarcinoma (LUAD), lung squamous cell carcinoma (LUSC), prostate adenocarcinoma (PRAD), thyroid carcinoma (THCA), and (UCEC) (**Figure 1A**). However, PIK3C2A expression was significantly increased in cholangiocarcinoma (CHOL), liver hepatocellular carcinoma (LIHC), and stomach adenocarcinoma (STAD) (**Figure 1A**). In addition, we retrieved the RNA-seq and clinical data for 33 cancers from the GDC website. Univariate COX analysis revealed that PIK3C2A is a protective factor for overall survival (OS), disease-free survival (DFS), and progression-free survival (PFS) only in KIRC (**Figure 1B**). As a result, we selected KIRC to further investigate the role of PIK3C2A in tumor progression.

### PIK3C2A expression in KIRC

**Figure 2:**
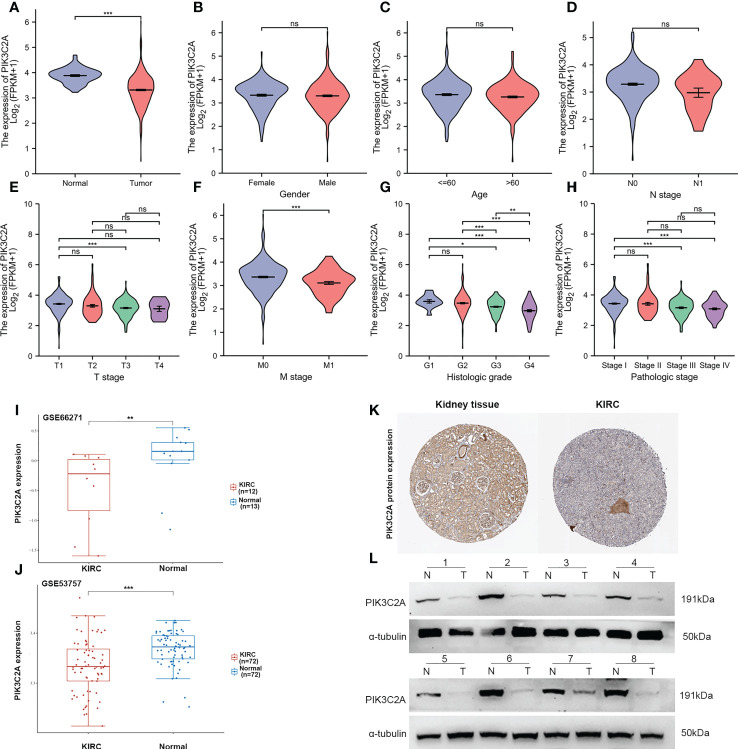
PIK3C2A expression status in KIRC. **(A)** The PIK3C2A expression in KIRC and normal tissues. **(B-H)** The expression of PIK3C2A in KIRC samples with different gender, ages, T stages, N stages, M stages, histologic grades, and pathologic stages. **(I, J)** Validation of PIK3C2A expression in KIRC and normal tissues in the GSE66271 and GSE52357 datasets. **(K)** The protein levels of PIK3C2A in KIRC and normal tissue in the Human Protein Atlas (Antibody HPA037641, 10X). **(L)** Protein expression of PIK3C2A in select KIRC tissues (T, n = 8) and neighboring non-tumor kidney tissues (N, n = 8). *p < 0.05, **p < 0.01, ***p < 0.001, ns, no significance.

Based on the TCGA database, PIK3C2A mRNA expression level was investigated in KIRC. The outcomes exhibited significantly lower mRNA levels of PIK3C2A in KIRC tissues than in normal tissues (**Figure 2A**). These findings were validated in the GEO database; the results demonstrated lower PIK3C2A expression levels in KIRC tissues than in normal tissues (**Figures 2I, J**). According to the Human Protein Atlas database (18), the PIK3C2A protein level was significantly lower in KIRC tissues compared with adjacent non-tumor kidney tissues (**Figure 2K**). PIK3C2A protein expression level was further evaluated in eight pairs of KIRC and neighboring non-tumor kidney tissues by western blot. The results demonstrated lower PIK3C2A protein levels in KIRC tissues than in neighboring non-tumor kidney tissues (**Figure 2L**). Furthermore, we investigated the expression changes of PIK3C2A in KIRC samples with different clinicopathologic features; the results revealed no significant difference in PIK3C2A expression in KIRC samples with varying gender, age, and N stages (**Figure 2B–D**). However, PIK3C2A expression decreased with the progression of the T stage, M stage, histologic grade, and pathologic stage (**Figures 2E–H**).

### PIK3C2A expression in scRNA data

**Figure 3:**
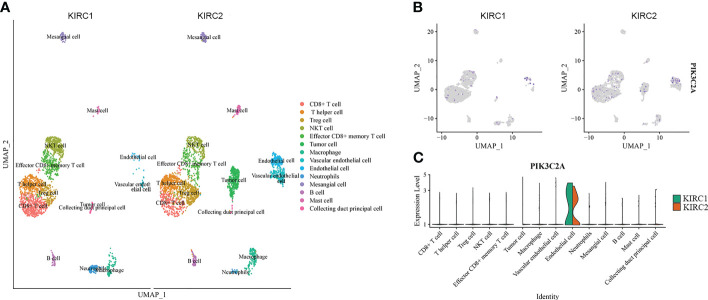
The expression of PIK3C2A in scRNA data. **(A)** Uniform manifold approximation and projection (UMAP) visualization of cells from the right (KIRC1) and left (KIRC2) KIRC tissues (KIRC2). **(B)** The expression pattern of PIK3C2A was plotted in the UMAP plot. **(C)** Expression level of PIK3C2A in different cell subgroups of KIRC tissues.

PIK3C2A expression in diverse cell subpopulations of KIRC was evaluated using scRNA-seq data. The dataset included 3568 cells from the right KIRC tissue (KIRC1) and 3575 cells from the left KIRC tissue (KIRC2). The data were subjected to UMAP analysis following quality control. Cells in KIRC were classified into 14 distinct categories, including CD8+ cell, T helper cell, Treg cell, NKT cell, effector CD8+ memory T cell, tumor cell, macrophage, vascular endothelial cell, endothelial cell, neutrophil, mesangial cell, B cell, mast cell and collecting duct principal cell, according to data on previously reported markers (**Figure 3A**). PIK3C2A-expressing cells were displayed on the UMAP plot, revealing that PIK3C2A was primarily expressed in endothelial cells (**Figure 3B**). Furthermore, we assessed the expression levels of PIK3C2A in each subgroup and discovered that it is expressed in various immune cells (**Figure 3C**). PIK3C2A expression in tumor cells was dramatically downregulated in the right KIRC tissue (KIRC1) (**Figure 3C**).

### Prognostic roles of PIK3C2A in KIRC

**Figure 4:**
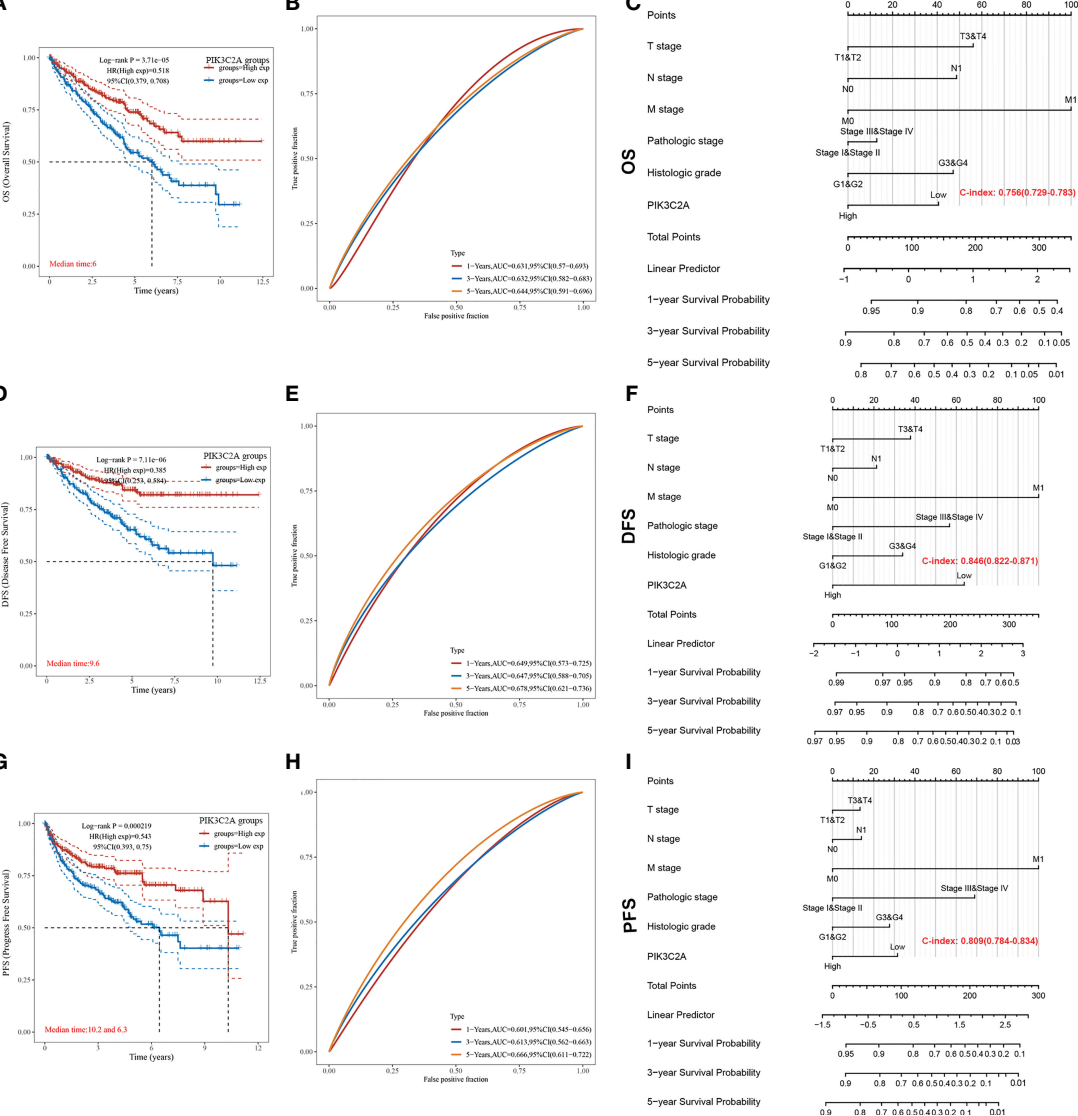
Estimating the predictive value of PIK3C2A for KIRC patients in the TCGA database. **(A)** Kaplan-Meier survival curve analysis for the Overall Survival of KIRC patients with high or low PIK3C2A expression. The differences between the two curves were determined by the two-sided log-rank test. **(B)** Time‐dependent ROC curve analysis of PIK3C2A for 1-year, 3- year and 5-year OS in KIRC patients. **(C)** The nomogram model for predicting the OS of KIRC. **(D)** Kaplan-Meier survival curve analysis for the Disease-Free Survival of KIRC patients with high or low PIK3C2A expression. The differences between the two curves were determined by the two-sided log-rank test. **(E)** Time‐dependent ROC curve analysis of PIK3C2A for 1-year, 3-year and 5-year DFS in KIRC patients. **(F)** The nomogram model for predicting the DFS of KIRC. **(G)** Kaplan-Meier survival curve analysis for the Progress Free Survival of KIRC patients with high or low PIK3C2A expression. The differences between the two curves were determined by the two-sided log-rank test. **(H)** Time‐dependent ROC curve analysis of PIK3C2A for 1-year, 3-year and 5-year PFS in KIRC patients. **(I)** The nomogram model for predicting the PFS of KIRC.

The Kaplan-Meier curve confirmed that the OS rate of the high-expression subset was significantly higher than that of the low-expression subset (*P*-value=3.71e-05, **Figure 4A**). Besides, the ROC curves were used to evaluate the predictive accuracy of PIK3C2A. PIK3C2A AUCs were 0.631, 0.632, and 0.644 at 1‐, 3‐ and 5‐year OS periods, respectively (**Figure 4B**). Similarly, the Kaplan Meier statistical analysis revealed that PIK3C2A may be a protective factor for the DFS rate (*P*-value=7.11e-06, **Figure 4D**) and PFS rate (*P*-value=2.19e-04, **Figure 4G**) of KIRC. The time-dependent ROC curves for DFS demonstrated that the 1-year, 3-year, and 5-year AUC values of PIK3C2A were 0.649%, 0.647%, and 0.678%, respectively (**Figure 4E**). The time-dependent ROC curves for PFS revealed a 1-year, 3-year, and 5-year AUC value of PIK3C2A of 0.601, 0.613, and 0.666, respectively (**Figure 4H**). These results demonstrated better predictive performance of PIK3C2A over time.

### Correlation of PIK3C2A with clinic-pathological properties of KIRC

The relationship between PIK3C2A and the clinical properties of KIRC was investigated using the TCGA database. The results demonstrated a correlation of PIK3C2A with the T stage (*p*-value<0.001), M stage (*p*-value=0.004), pathologic stage (*p*-value<0.001), and histologic grade (*p*-value<0.001) (**Table 1**). These findings confirmed that PIK3C2A expression decreases following the progression of the T stage, M stage, pathologic stage, and histologic grade of KIRC.

**Table 1:** Correlations of PIK3C2A expression with the clinicopathological features of KIRC.

Characteristic	levels	Low expression of PIK3C2A	High expression of PIK3C2A	*p*-value
n		269	270	
T stage, n (%)	T1	112 (20.8%)	166 (30.8%)	**< 0.001**
	T2	40 (7.4%)	31 (5.8%)	
	T3	111 (20.6%)	68 (12.6%)	
	T4	6 (1.1%)	5 (0.9%)	
N stage, n (%)	N0	121 (47.1%)	120 (46.7%)	0.238
	N1	11 (4.3%)	5 (1.9%)	
M stage, n (%)	M0	201 (39.7%)	227 (44.9%)	**0.004**
	M1	51 (10.1%)	27 (5.3%)	
Pathologic stage, n (%)	Stage I	110 (20.5%)	162 (30.2%)	**< 0.001**
	Stage II	29 (5.4%)	30 (5.6%)	
	Stage III	74 (13.8%)	49 (9.1%)	
	Stage IV	54 (10.1%)	28 (5.2%)	
Primary therapy outcome, n (%)	PD	7 (4.8%)	4 (2.7%)	0.051
	SD	0 (0%)	6 (4.1%)	
	PR	1 (0.7%)	1 (0.7%)	
	CR	52 (35.4%)	76 (51.7%)	
Age, n (%)	<=60	124 (23%)	145 (26.9%)	0.093
	>60	145 (26.9%)	125 (23.2%)	
Histologic grade, n (%)	G1	3 (0.6%)	11 (2.1%)	**< 0.001**
	G2	96 (18.1%)	139 (26.2%)	
	G3	112 (21.1%)	95 (17.9%)	
	G4	54 (10.2%)	21 (4%)	
Serum calcium, n (%)	Elevated	6 (1.6%)	4 (1.1%)	0.399
	Low	98 (26.8%)	105 (28.7%)	
	Normal	84 (23%)	69 (18.9%)	
Hemoglobin, n (%)	Elevated	2 (0.4%)	3 (0.7%)	0.573
	Low	142 (30.9%)	121 (26.4%)	
	Normal	94 (20.5%)	97 (21.1%)	
Laterality, n (%)	Left	131 (24.3%)	121 (22.5%)	0.391
	Right	137 (25.5%)	149 (27.7%)	

Significant results (P<0.05) are given in bold.

### Independence of PIK3C2A and other clinical characteristics

The independence of PIK3C2A from other clinicopathological characteristics of KIRC was determined using univariate and multivariate Cox regression analysis. Results from the univariate Cox regression analysis of KIRC patients revealed that PIK3C2A expression, T stage, N stage, M stage, histologic grade, and pathologic stage were all significant predictors of OS, DFS, and PFS (**Table 2**). A multivariate Cox regression analysis revealed that PIK3C2A expression and M stage are independent prognostic variables for OS, DFS, and PFS in KIRC patients (**Table 2**). In this view, PIK3C2A expression was suggested as a clinically promising biomarker for predicting patient outcomes in KIRC. Using the aforementioned variables, we developed nomograms to forecast the OS, DFS, and PFS of KIRC (**Figures 4C, F, I**). The C-index in the nomogram model for OS was 0.756, 0.846 for DFS, and 0.809 for PFS.

**Table 2:** Univariate and multivariate Cox regression analysis in the TCGA database.

Characteristics	Total(N)	OS				
		Univariate analysis			Multivariate analysis	
		Hazard ratio (95% CI)	P value		Hazard ratio (95% CI)	P value
T stage (T3&T4 vs. T1&T2)	539	3.228 (2.382-4.374)	**<0.001**		1.777 (0.770-4.100)	0.178
N stage (N1 vs. N0)	257	3.453 (1.832-6.508)	**<0.001**		1.593 (0.798-3.180)	0.187
M stage (M1 vs. M0)	506	4.389 (3.212-5.999)	**<0.001**		2.667 (1.579-4.506)	**<0.001**
Pathologic stage (Stage III&Stage IV vs. Stage I&Stage II)	536	3.946 (2.872-5.423)	**<0.001**		1.131 (0.440-2.907)	0.798
Histologic grade (G3&G4 vs. G1&G2)	531	2.702 (1.918-3.807)	**<0.001**		1.579 (0.951-2.623)	0.078
PIK3C2A (High vs. Low)	539	0.480 (0.351-0.658)	**<0.001**		0.634 (0.404-0.993)	**0.047**
				DFS		
Characteristics	Total(N)	Univariate analysis			Multivariate analysis	
		Hazard ratio (95% CI)	**P value**	Hazard ratio (95% CI)	P value
T stage (T3&T4 vs. T1&T2)	528	5.542 (3.652-8.411)	**<0.001**		1.729 (0.724-4.129)	0.218
N stage (N1 vs. N0)	255	3.852 (1.825-8.132)	**<0.001**		1.328 (0.610-2.892)	0.474
M stage (M1 vs. M0)	495	9.108 (6.209-13.361)	**<0.001**		4.006 (2.192-7.321)	**<0.001**
Pathologic stage (Stage III&Stage IV vs. Stage I&Stage II)	525	9.835 (5.925-16.325)	**<0.001**		2.240 (0.731-6.863)	0.158
Histologic grade (G3&G4 vs. G1&G2)	520	4.793 (2.889-7.952)	**<0.001**		1.609 (0.806-3.213)	0.178
PIK3C2A (High vs. Low)	528	0.349 (0.228-0.534)	**<0.001**		0.389 (0.209-0.722)	**0.003**
				PFS		
Characteristics	Total(N)	Univariate analysis			Multivariate analysis	
		Hazard ratio (95% CI)	**P value**	Hazard ratio (95% CI)	P value
T stage (T3&T4 vs. T1&T2)	537	4.522 (3.271-6.253)	**<0.001**		1.261 (0.604-2.636)	0.537
N stage (N1 vs. N0)	256	3.682 (1.891-7.167)	**<0.001**		1.211 (0.600-2.444)	0.593
M stage (M1 vs. M0)	504	8.968 (6.464-12.442)	**<0.001**		4.480 (2.606-7.700)	**<0.001**
Pathologic stage (Stage III&Stage IV vs. Stage I&Stage II)	534	6.817 (4.770-9.744)	**<0.001**		2.782 (1.115-6.943)	0.028
Histologic grade (G3&G4 vs. G1&G2)	529	3.646 (2.503-5.310)	**<0.001**		1.498 (0.884-2.539)	0.133
PIK3C2A (High vs. Low)	537	0.504 (0.364-0.698)	**<0.001**		0.578 (0.352-0.950)	**0.030**

Significant results (P<0.05) are given in bold.

### *In vitro* PIK3C2A downregulation promotes KIRC cell proliferation

**Figure 5:**
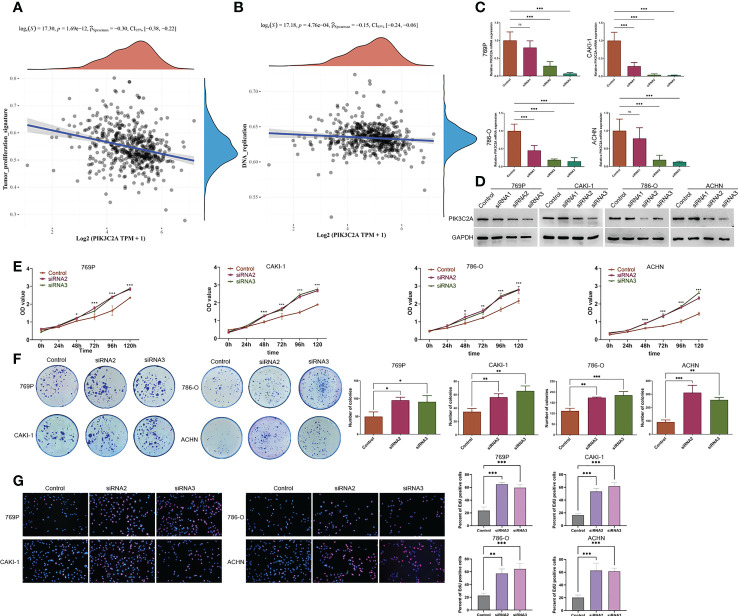
A decrease in PIK3C2A levels facilitated cell growth in KIRC cells. **(A, B)** The linear relationship of PIK3C2A expression with tumor proliferation signature and DNA replication pathway by Spearman’s correlation. **(C, D)**The levels of PIK3C2A mRNA and protein were significantly inhibited by siRNA2 and siRNA3. **(E)** CCK8 assay was used to detect cell proliferation in siRNA2- and siRNA3-transfected 769P,CAKI-1 786-O and ACHN cells. *P < 0.05, ***P < 0.001. **(F)** Using the colony-forming test, the colony-forming ability of 769P,CAKI-1 786-O and ACHN cells treated with siRNA2 and siRNA3 was evaluated. The number of colonies was counted and compared in the diagrams; significance was determined using one-way ANOVA. *P < 0.05, **P < 0.01, ***P < 0.001. **(G)** EdU assays were performed to determine the cell proliferation ability of si-PIK3C2A transfected cells. The ratio of EdU-positive cells was calculated and compared in the diagrams; significance was determined using one-way ANOVA. **P < 0.01, ***P < 0.001. ns, no significance.

Having found that PIK3C2A expression decreased in KIRC tissues, we analyzed the influence of PIK3C2A on the cellular biology of KIRC cells. Firstly, we assessed the relationship between PIK3C2A and tumor-related signal pathways using ssGSEA. According to the results, PIK3C2A expression was negatively correlated with tumor proliferation signature and DNA replication pathways (Spearman’s correlation value = -0.30 and -0.15, respectively, *P*<0.001; **Figures 5A, B**). Subsequently, PIK3C2A expression in KIRC cell lines was blocked using small interfering RNAs (siRNAs). Real-time PCR and Western blotting analyses revealed that siRNA2 and siRNA3 could significantly decrease PIK3C2A expression; as such, they were selected for further functional tests (**Figures 5C, D**). CCK8 detection results demonstrated that inhibiting PIK3C2A expression significantly promoted the proliferation of 769P, CAKI-1, 786-O, and ACHN cells (**Figure 5E**). Inhibiting the expression of PIK3C2A significantly increased the colony-forming ability of KIRC cells (**Figure 5F**). The EdU assay revealed a significantly higher proportion of EdU-positive cells in the PIK3C2A knockdown groups than in the control groups (**Figure 5G**). These results confirmed that PIK3C2A knockdown resulted in significantly increased KIRC cell proliferation *in vitro*.

### PIK3C2A alteration in KIRC induces changes in genes and biological processes

**Figure 6:**
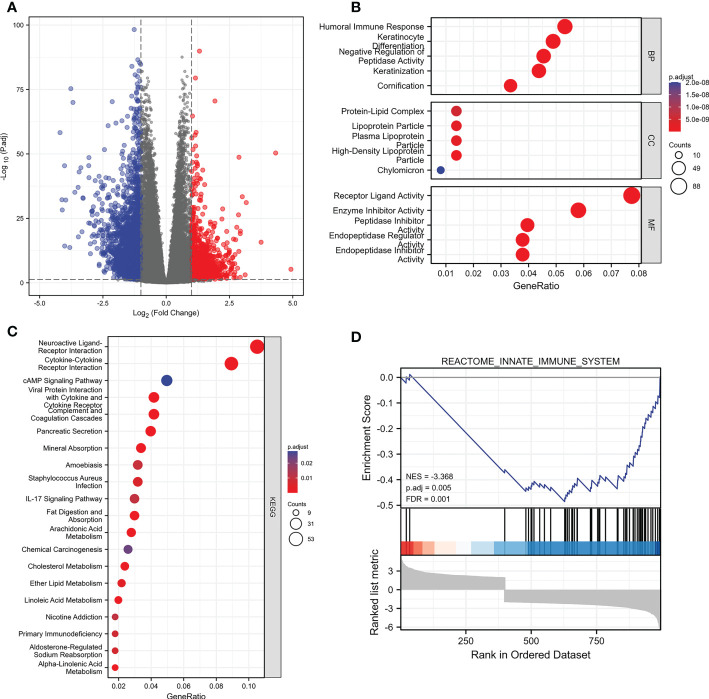
The changes of genes and biological processes caused by the PIK3C2A alteration in KIRC. **(A)** The volcano plot shows the differential expression genes between PIK3C2A high-expression and low-expression groups in KIRC. **(B, C)** The bubble charts visualize the results of GO term and KEGG pathway enrichment analysis of the differential expression genes. **(D)** The GSEA results of the differential expression genes.

We split the samples of the TCGA dataset into high-expression and low-expression groups based on the median value of PIK3C2A expression to determine gene alterations caused by PIK3C2A modifications. Following that, DEGs were identified using the DESeq2 Bioconductor package, with cut-offs set at a log2foldchange (log2FC) >1 and an adjusted *P*-value (adj. *P*) < 0.05. The results revealed that 1039 DEGs were up-regulated in the high-expression group, while 2815 PCGs were down-regulated in the low-expression group (**Figure 6A**). These DEGs were evaluated using the ClusterProfiler package (version: 3.18.0) in R to unravel the underlying functions and pathways. **Figures 6B, C** depict the distribution of these DEGs across molecular biological roles (BP), functions (MF), cellular components (CC), and KEGG pathways. The Gene Set Enrichment Analysis (GSEA) was used to verify the potential pathways activated or inhibited due to PIK3C2A alterations in KIRC. The Reactome innate immune system was the most significantly enriched signaling pathway (NSE = -3.368, adjusted *P*-value=0.005; **Figure 6D**), suggesting that PIK3C2A alterations may contribute to KIRC-related immunological dysfunction.

### Effects of PIK3C2A on the immune infiltration level in KIRC

**Figure 7:**
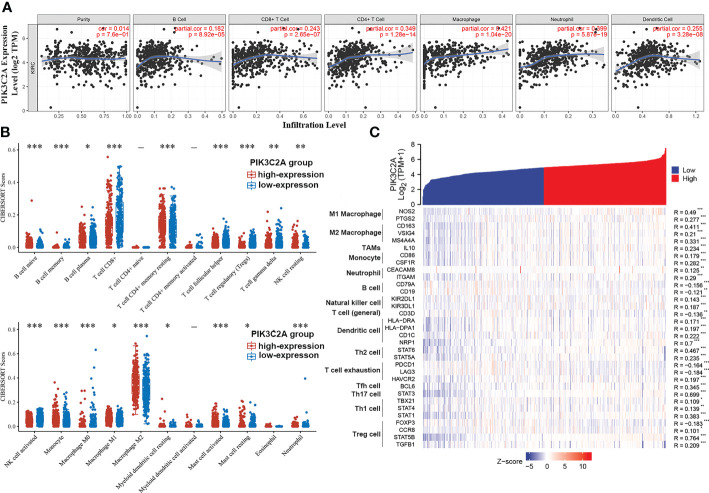
The correlation of PIK3C2A expression with immune infiltration levels in KIRC. **(A)** PIK3C2A expression has no relation with tumor purity and a significant positive correlation with infiltrating ranks of B cell, CD8+ T cell, CD4+ T cell, macrophage, neutrophil, and dendritic cell. **(B)** The immune score distribution of 22 immune cell subtypes in the PIK3C2A high-expression and low-expression group of KIRC. The horizontal axis represents different groups of samples. The vertical axis represents the gene expression distribution, where different colors represent different groups, and the significance of the two groups was tested by the Wilcox test. Asterisks denote levels of significance (*p < 0.05, **p < 0.01, ***p < 0.001). **(C)** The heatmap displays the relationship between PIK3C2A expression and gene markers of immune cells in KIRC (*p < 0.05, **p < 0.01, ***p < 0.001).

According to the analysis in the TIMER database, PIK3C2A was positively correlated with the infiltration degrees of B cells (R = 0.182, *P* = 8.92E–05), CD8+ T cells (R = 0.243, *P* = 2.65E–07), CD4+ T cells (R = 0.349, *P* =1.28E−14), macrophages (R = 0.421, *P* = 1.04E–20), neutrophils (R = 0.399, *P* = 5.87E–19), and dendritic cells (R = 0.255, *P* = 3.28E−08) in KIRC (**Figure 7A**). In addition, using the CIBERSORT algorithm, we evaluated the correlation between PIK3C2A status and 22 immune fractions, uncovering a widespread immune infiltration pattern of KIRC. The results revealed a higher infiltration degree of B cell native (adjusted *p*-value= 6.49E-10), B cell plasma (adjusted *p*-value=4.88E-02), T cell CD4+ memory resting (adjusted *p*-value = 8.92E-04), NK cell resting (adjusted *p*-value= 1.29E-02), Monocyte (adjusted *p*-value= 3.91E-08), Macrophage M1 (adjusted *p*-value= 1.69E-02), Macrophage M2 (adjusted *p*-value=4.64E-04), Myeloid dendritic cell resting (adjusted *p*-value= 3.84E-02), Mast cell activated (adjusted *p*-value= 1.62E-05) and Neutrophil (adjusted *p*-value = 2.72E-05) in the PIK3C2A high-expression group than in the PIK3C2A low-expression group (**Figure 7B**). On the contrary, the infiltration degree of B cell memory (adjusted *p*-value= 3.05E-10), T cell CD8+ (adjusted *p*-value= 9.10E-06), T cell follicular helper (adjusted *p*-value= 1.33E-10), T cell regulatory (adjusted *p*-value= 2.56E-15), T cell gamma delta (adjusted *p*-value=3.59E-03), NK cell activated (adjusted *p*-value= 2.48E-08), Macrophage M0 (adjusted *p*-value= 1.16E-06) and Mast cell resting (adjusted *p*-value= 1.65E-02) was lower in the PIK3C2A high-expression group than in the PIK3C2A low-expression group (**Figure 7B**). These results highlight a crucial role for PIK3C2A in regulating the immune infiltration microenvironment of KIRC.

### Correlation between PIK3C2A and immune markers

Based on data from the TIMER and TCGA databases, we analyzed the correlations between PIK3C2A and a wide range of immunological indicators in KIRC to shed light on the potential links between this gene and invading immune cells. The PIK3C2A level was found to be closely correlated with 37 out of 57 immune cell markers in KIRC, after adjusting for the purity in the TIMER database (**Supplementary Table 1**). The TCGA database analysis revealed that NOS2 and PTGS2 of the M1 phenotype (**Figure 7C**), CD163, VSIG4, and MS4A4A of the M2 phenotype (**Figure 7C**), IL10 of TAMs (**Figure 7C**), CD86 and CSF1R of monocytes (**Figure 7C**), CEACAM8 and ITGAM of neutrophils (**Figure 7C**) were significantly correlated with PIK3C2A expression in KIRC. The GEPIA database was used to determine whether the association between the two immune cell types and PIK3C2A expression in KIRC and comparable normal tissues was distinct. The analysis yielded comparable findings to TCGA results; the correlations between PIK3C2A expression and macrophages or neutrophils were quite robust, with 12 out of 14 immune cell markers; However, 2 out of 14 immune cell indicators correlated significantly with PIK3C2A expression in renal tissues (**Table 3**). These data suggested a potential regulatory role of PIK3C2A on macrophage polarization and neutrophil infiltration in KIRC, but this activity was extremely poor in normal tissues.

**Table 3:** Correlation analysis between PIK3C2A and gene markers of immune cells in GEPIA.

Cell type	Gene markers		KIRC		
		Tumor		Normal	
		R	P	R	P
TAM	CCL2	0.085	0.051	0.19	0.11
	CD68	0.27	**4.30E-10**	-0.046	0.7
	IL10	0.27	**2.30E-10**	0.11	0.37
M1 Macrophage	INOS (NOS2)	0.52	**6.20E-37**	0.2	0.095
	IRF5	0.083	0.06	0.31	**9.10E-03**
	COX2(PTGS2)	0.28	**3.90E-11**	0.39	**6.00E-04**
M2 Macrophage	CD163	0.26	**2.10E-09**	0.068	0.57
	VSIG4	0.22	**5.10E-07**	0.1	0.39
	MS4A4A	0.36	**9.70E-18**	0.04	0.74
Monocyte	CD86	0.24	**2.00E-08**	0.085	0.48
	CD115 (CSF1R)	0.32	**8.20E-14**	0.17	0.16
Neutrophils	CD66b (CEACAM8)	0.13	**2.30E-03**	0.23	0.056
	CD11b (ITGAM)	0.33	**1.60E-14**	0.2	0.094
	CCR7	0.11	**1.50E-02**	0.084	0.48

Significant results (P<0.05) are given in bold.

Furthermore, there was a considerable variation in the correlation of PIK3C2A expression with B cells, natural killer cells, dendritic cells (DCs), T regulatory cells (Tregs), T helper cells (Th cells), and T cell exhaustion in KIRC or normal tissues. The results demonstrated that the markers of B cells, including CD19 and CD79A (**Figure 7C**), are negatively correlated with PIK3C2A expression. For natural killer cells, we found a positive correlation of PIK3C2A with KIR2DL1 and KIR2DL3 in KIRC but not in normal tissues (**Figure 7C**). PIK3C2A was highly relevant to DC infiltration in KIRC (**Figure 7C**). Further research is warranted to establish whether PIK3C2A has dual effects on cancer immunity. PIK3C2A was closely correlated with T cell exhaustion and Treg; PIK3C2A was positively linked to CCR8, STAT5B, and TGFB1, and negatively related to FOXP3, PDCD1, and LAG3 (**Figure 7C**). Also, we found a strong correlation between PIK3C2A and biomarkers of T helper cells, including Th1, Th2, Tfh, and Th17 cells (**Figure 7C**). These add to the evidence that PIK3C2A is strongly linked to immune infiltration in KIRC and may play a dualistic role in tumor immune responses.

### PIK3C2A networks of genes

**Figure 8:**
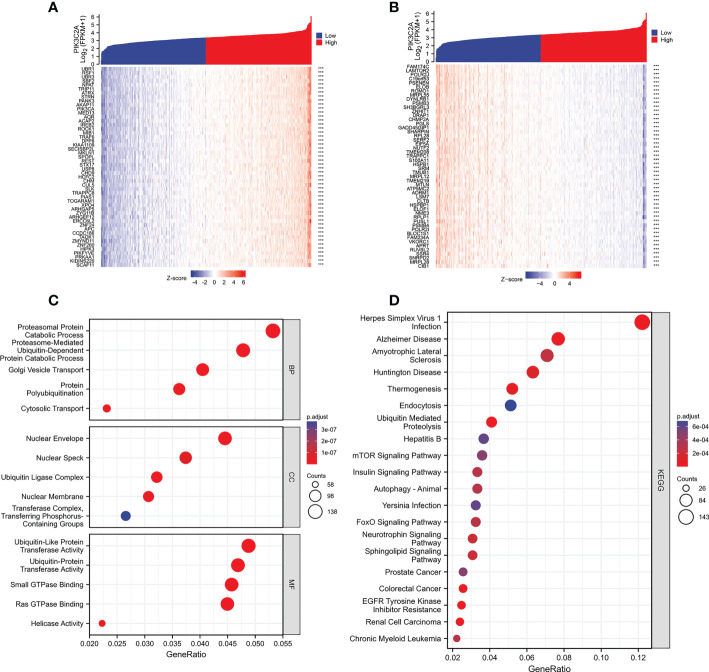
Functional enrichment analysis of the PCGs associated with PIK3C2A. **(A)** The heatmap exhibits the top 50 genes positively related to PIK3C2A. **(B)** The heatmap indicates the top 50 genes negatively associated with PIK3C2A. **(C, D)** The bubble charts visualize the results of GO term and KEGG pathway enrichment analysis of the PCGs co-expressed with PIK3C2A.

The correlation of PIK3C2A with PCGs was evaluated using Spearman correlation analysis to determine the precise mechanism of PIK3C2A in KIRC carcinogenesis. The results revealed that 2345 PCGs and 466 PCGs were positively and negatively correlated with PIK3C2A, respectively (Spearman’s correlation coefficient>0.5, adjusted *p*-value<0.05); the top 50 positively correlated and negatively correlated PCGs are shown in **Figures 8A, B**, respectively. Functional enrichment analysis demonstrated that PIK3C2A correlated PCGs were primarily enriched in 245 gene ontology (GO) terms (adjust *p*-value<0.001; **Supplementary Table 2**) and 22 Kyoto Encyclopedia of Genes and Genomes (KEGG) pathways (adjust *p*-value<0.001; **Supplementary Table 3**); the typical GO terms and KEGG pathways are displayed in **Figures 8C, D**, respectively.

## Discussion

There is growing evidence that tumor cells can escape killing by the innate body’s anti-tumor mechanisms. Furthermore, the dynamic interaction between malignancies and the immune system is an important factor influencing the development of treatment resistance in cancer cells (19). Human tumor tissues contain a wide range of immune cell types known as tumor-infiltrating immune cells, which have been shown to affect the prognosis of various cancers and to be potential immunotherapy targets. The infiltrating immune cell-based immunological score is considered an independent risk factor affecting cancer prognosis (20, 21). Significant and rapid progress has been made in the last few decades in terms of understanding how the immune system control cancer progression. Parallel to these studies, cancer immunotherapies have been created to activate the immune system and hence induce potent anti-tumor responses (22). Recent advances in the development of cancer immunotherapies have improved the way cancer treated. Tumor recurrence and severe side effects, such as autoimmunity and nonspecific inflammation, are common problems reported in many patients (23, 24). As such, a synthetical and translational analysis is required to characterize the tumor immunophenotypes and predict the anti-tumor immune responses, as well as reveal the underlying molecular mechanisms of cancer immunity.

To date, no comprehensive study has been conducted to evaluate the role of PIK3C2A in cancer, and characterize its prognostic landscape. Therefore, several data sources were screened to fully comprehend the functions of PIK3C2A in cancer. According to the TIMER analysis of TCGA data, PIK3C2A expression was discovered to be significantly overexpressed in KIRC compared to adjacent normal tissues. The expression of PIK3C2A was also reduced as the T stage, M stage, pathologic stage, and histologic grade of KIRC increased. Further analysis of scRNA data showed that the PIK3C2A enrichment was higher in endothelial cells than in KIRC cells. PIK3C2A was also found to be correlated with the prognosis of numerous cancers, and predicted favorable OS, DFS, and PFS in KIRC. This was also confirmed by the time-dependent ROC curve analysis. According to the results of *in vitro* functional experiments, downregulation of PIK3C2A expression significantly increased the proliferative capacities of KIRC cells. For these results, an increase in PIK3C2A level enhanced the ability of the cells to inhibit oncogenesis and progression of KIRC. Moreover, GO functional results showed that the biological process of the humoral immune response was enriched in PIK3C2A-related DEGs. These DEGs exhibited high enrichment in signal pathways involved in the Reactome’s innate immune system as indicated by the GSEA results. Moreover, the results of scRNA data indicated that PIK3C2A was enriched in several types of immune cells. Collectively, these results suggest that PIK3C2A’s influence on the immune system may be responsible for its pleiotropic involvement in the development of KIRC.

Immunocyte populations are the primary components in the tumor immune network and have a significant impact on the development and incidence of malignancies. In this work, the relationships between PIK3C2A and tumor immunocyte infiltration were investigated by relying on the TIMER database. It was found that PIK3C2A influenced immunocyte infiltration in many cancers, particularly KIRC. There was a strong positive relationship between PIK3C2A level and infiltration degree of macrophages, neutrophils, CD4+ T cells, DCs, and B cells. Additionally, the interrelation between PIK3C2A and the biomarkers of immunocytes suggested that PIK3C2A was involved in the modulation of cancer immunity in KIRC. First, PIK3C2A was not related to tumor purity in KIRC, indicating that PIK3C2A was both expressed in malignant cells and the tumor microenvironment. Nevertheless, macrophages, master regulators of inflammation and antigen-presenting had the highest correlation coefficient with PIK3C2A compared with other immune cells. After adjustment for tumor purity, M1 and M2 macrophage markers exhibited strong correlations with PIK3C2A expression. These results demonstrated the underlying modulatory role of PIK3C2A in the polarized alternatively of tumor-associated macrophages (TAM) in KIRC.

In addition, an inverse correlation was found between the level of PIK3C2A and the gene markers of T-cell exhaustion. Cancer cells rely heavily on exhausted T cells evade the immune system because these cells have low cytokine levels and effector function (25). In addition, numerous studies have demonstrated that inhibition of the causes of T-cell exhaustion represents a novel therapeutic approach for improving reactive antitumor immunity. Blocking antibodies for PD-1/PD-L and LAG-3 are effective therapeutic methods for activating dormant T cells (26, 27). Thus, upregulating PIK3C2A expression may be a feasible combination strategy to bolster the antineoplastic effectiveness of checkpoint inhibitors by decreasing levels of T cell exhaustion and reversing T cell depletion.

Further analysis revealed strong relationships between PIK3C2A levels and Treg genetic markers. Foxp3 is the most prominent transcription factor in Treg, and its expression is inversely related to that of PIK3C2A. Although it plays an important in escape mechanisms against autoimmunity, FOXP3+ Treg dampens tumor immunity (28). Moreover, the CIBERSORT results revealed that the infiltration degree of Treg was negatively correlated with PIK3C2A expression. Therefore, we speculate that PIK3C2A can transform Tregs into proinflammation cells which have anti-cancer-promoting utility by decreasing the expression of FOXP3. Besides, PIK3C2A is associated with several markers of T-helper subpopulations. T helper cells belong to the CD4+ T lymphocytes, which participate in adaptive immune responses (29). To properly orchestrate immune responses against tumor cells, a healthy balance of Th subtypes is required (30). Studies have shown that T helper cells have varying effects on cancer clinical outcomes; with elevated Th17 biomarkers predicting poor outcomes, whereas those with elevated Th1 biomarkers have longer DFS (31). In conclusion, PIK3C2A participates in the recruitment and modulation of immunocyte infiltration in KIRC, suggesting that adjusting PIK3C2A expression may be a useful therapeutic strategy to restore an effective anti-tumor immune response.

Moreover, according to the function-rich study, the genes co-expressed with PIK3C2A were primarily associated with ubiquitin, which is the essential mechanism through which its regulates the translation of innate immune response signals (32). Moreover, KEGG enrichment analysis revealed that these genes were enriched in mTOR signaling. Thus, it is now considered to regulate several features of the immunosuppressive tumor microenvironment (33). In addition, the KEGG enrichment results demonstrated that these genes were highly enriched in the insulin signaling pathway. Previous research has shown that insulin stimulation can activate PIK3C2A and that the activated PIK3C2A in turn stimulates the synthesis of PIP2 and PIP3 (34). Consequently, the relationship between PIK3C2A, ubiquitin, insulin, and the mTOR signaling pathway may be an important modulator of tumor immunity.

In conclusion, PIK3C2A may be an independent prognostic marker and it regulates the infiltration of immune cells in many types of malignancies. Specifically, in KIRC, PIK3C2A overexpression is associated with a better prognosis and increased immunocyte infiltration. Moreover, KIRC’s proliferative capacities are altered by aberrant PIK3C2A expression. Further research is needed to develop anti-tumor agents based on these results.

## Data availability statement

The original contributions presented in the study are included in the article/**Supplementary Material**. Further inquiries can be directed to the corresponding authors.

## Author contributions

DM and YY contributed to the conception and design of the study. CQ, SL, and QW drafted the manuscript. CQ, SL, SZ, XX, JH, XY, and ZW collected and analyzed the data. DM and YY revised the manuscript. All authors contributed to the manuscript revision and read and approved the submitted version.
