# Supplementary material for: PIK3C2A is a prognostic biomarker that is linked to immune infiltrates in kidney renal clear cell carcinoma
Source: Front Immunol. 2023 Mar 30;14:1114572. doi: 10.3389/fimmu.2023.1114572 (PMC10098324; doi:10.3389/fimmu.2023.1114572)
Supplement: Supplementary file 1 [file Table_1.docx]

| **Description**  **Supplementary table 1 Correlation analysis between PIK3C2A and gene markers of immune cells in TIMER.** | **Gene markers** |  |  |  | **KIRC** |  |  |
| --- | --- | --- | --- | --- | --- | --- | --- |
|  |  |  | **None** | |  | **Purity** | |
|  |  |  | **Cor** | **P** |  | **Cor** | **P** |
| CD8+ T cell | CD8A |  | -0.03438 | 0.428300859 |  | -0.03322 | 0.476794604 |
|  | CD8B |  | -0.09554 | **0.027418863** |  | -0.09697 | **0.037398945** |
| T cell (general) | CD3D |  | -0.15811 | **0.000247458** |  | -0.16795 | **0.000292279** |
|  | CD3E |  | -0.10186 | **0.018659085** |  | -0.10797 | **0.020408669** |
|  | CD2 |  | -0.05905 | 0.173435179 |  | -0.05957 | 0.20169638 |
| B cell | CD19 |  | -0.14266 | **0.000957258** |  | -0.13567 | **0.00351722** |
|  | CD79A |  | -0.15117 | **0.000461742** |  | -0.15691 | **0.000722703** |
| Monocyte | CD86 |  | 0.126522 | **0.003434663** |  | 0.134521 | **0.003808504** |
|  | CD115 (CSF1R) | | 0.207252 | **1.39E-06** |  | 0.205419 | **8.75E-06** |
| TAM | CCL2 |  | 0.031657 | 0.465807776 |  | 0.081505 | 0.080440726 |
|  | CD68 |  | 0.051912 | 0.231508277 |  | 0.034928 | 0.454380104 |
|  | IL10 |  | 0.179017 | **3.23E-05** |  | 0.173141 | **0.000187229** |
| M1 Macrophage | INOS (NOS2) |  | 0.477294 | **1.12E-31** |  | 0.481745 | **3.67E-28** |
|  | IRF5 |  | -0.01784 | 0.681097321 |  | -0.00681 | 0.883987625 |
|  | COX2(PTGS2) |  | 0.226123 | **1.32E-07** |  | 0.25027 | **5.16E-08** |
| M2 Macrophage | CD163 |  | 0.396932 | **1.47E-21** |  | 0.385808 | **8.24E-18** |
|  | VSIG4 |  | 0.159762 | **0.000212599** |  | 0.13575 | **0.003496976** |
|  | MS4A4A |  | 0.280355 | **4.40E-11** |  | 0.28432 | **5.07E-10** |
| Neutrophils | CD66b (CEACAM8) | | 0.17317 | **5.85E-05** |  | 0.168343 | **0.000282759** |
|  | CD11b (ITGAM) |  | 0.221671 | **2.34E-07** |  | 0.222769 | **1.36E-06** |
|  | CCR7 |  | 0.03599 | 0.406984168 |  | 0.035531 | 0.446623789 |
| Natural killer cell | KIR2DL1 |  | 0.121854 | **0.004845622** |  | 0.09416 | **0.04331108** |
|  | KIR2DL3 |  | 0.099233 | **0.021948353** |  | 0.080816 | 0.083040998 |
|  | KIR2DL4 |  | -0.1064 | **0.013982658** |  | -0.1202 | **0.00979247** |
|  | KIR3DL1 |  | 0.142664 | **0.000957151** |  | 0.127343 | **0.006183319** |
|  | KIR3DL2 |  | -0.02185 | 0.61477113 |  | -0.02547 | 0.58549247 |
| Dendritic cell | HLA-DPB1 |  | 0.0614 | 0.156913312 |  | 0.053695 | 0.249905475 |
|  | HLA-DQB1 |  | 0.04852 | 0.263485951 |  | 0.050343 | 0.28074233 |
|  | HLA-DRA |  | 0.149612 | **0.000529281** |  | 0.149776 | **0.001258375** |
|  | HLA-DPA1 |  | 0.163006 | **0.000156981** |  | 0.174967 | **0.000159575** |
|  | BDCA-1(CD1C) |  | 0.223477 | **1.85E-07** |  | 0.242102 | **1.42E-07** |
|  | BDCA-4(NRP1) |  | 0.674304 | **6.15E-72** |  | 0.688252 | **5.71E-66** |
|  | CD11c (ITGAX) |  | -0.00715 | 0.869276495 |  | -0.00312 | 0.946745834 |
| Th1 | T-bet (TBX21) |  | 0.107424 | **0.013085103** |  | 0.110596 | **0.017528469** |
|  | STAT4 |  | 0.10221 | **0.01825796** |  | 0.118304 | **0.011017568** |
|  | STAT1 |  | 0.333751 | **2.47E-15** |  | 0.339495 | **6.72E-14** |
|  | IFN-γ (IFNG) |  | -0.08336 | 0.054422619 |  | -0.0904 | 0.052429688 |
|  | TNF-α (TNF) |  | 0.090421 | **0.036897396** |  | 0.084602 | 0.069554394 |
| Th2 | GATA3 |  | -0.02601 | 0.549129556 |  | -0.01042 | 0.823423202 |
|  | STAT6 |  | 0.457769 | **5.77E-29** |  | 0.434985 | **1.05E-22** |
|  | STAT5A |  | 0.206597 | **1.51E-06** |  | 0.220289 | **1.79E-06** |
|  | IL13 |  | -0.06715 | 0.121551765 |  | -0.01582 | 0.734748777 |
| Tfh | BCL6 |  | 0.281474 | **3.66E-11** |  | 0.285955 | **4.00E-10** |
|  | IL21 |  | -0.02511 | 0.56298209 |  | -0.03416 | 0.46434895 |
| Th17 | STAT3 |  | 0.68734 | **9.23E-76** |  | 0.702996 | **6.10E-70** |
|  | IL17A |  | -0.04585 | 0.290722555 |  | -0.00837 | 0.857817005 |
| Treg | FOXP3 |  | -0.17409 | **5.33E-05** |  | -0.16866 | **0.000275176** |
|  | CCR8 |  | 0.101745 | **0.018795899** |  | 0.130011 | **0.005178038** |
|  | STAT5B |  | 0.788195 | **4.94E-114** |  | 0.7966 | **2.15E-102** |
|  | TGFβ (TGFB1) |  | 0.155769 | **0.000306419** |  | 0.156278 | **0.000759813** |
| T cell exhaustion | PD-1 (PDCD1) |  | -0.18674 | **1.43E-05** |  | -0.18638 | **5.67E-05** |
|  | CTLA4 |  | -0.06933 | 0.109875201 |  | -0.06069 | 0.19338415 |
|  | LAG3 |  | -0.18979 | **1.03E-05** |  | -0.19688 | **2.07E-05** |
|  | TIM-3 (HAVCR2) | | 0.189599 | **1.05E-05** |  | 0.186777 | **5.46E-05** |
|  | GZMB |  | -0.09771 | **0.024082118** |  | -0.11312 | **0.015103273** |
