# Supplementary material for: PIK3C2A is a prognostic biomarker that is linked to immune infiltrates in kidney renal clear cell carcinoma
Source: Front Immunol. 2023 Mar 30;14:1114572. doi: 10.3389/fimmu.2023.1114572 (PMC10098324; doi:10.3389/fimmu.2023.1114572)
Supplement: Supplementary file 2 [file Table_2.docx]

| **Supplementary table 2** The results of GO term enrichment analysis of the PCGs co-expressed with PIK3C2A. | | | | | | | |
| --- | --- | --- | --- | --- | --- | --- | --- |
| ONTOLOGY | ID | Description | GeneRatio | BgRatio | pvalue | p.adjust | qvalue |
|  |  |  |  |  |  |  |  |
| BP | GO:0010498 | proteasomal protein catabolic process | 138/2594 | 477/18670 | 4.0112E-18 | 2.3987E-14 | 2.0415E-14 |
| BP | GO:0043161 | proteasome-mediated ubiquitin-dependent protein catabolic process | 124/2594 | 419/18670 | 2.9921E-17 | 8.9464E-14 | 7.6141E-14 |
| BP | GO:0016482 | cytosolic transport | 60/2594 | 158/18670 | 3.6701E-14 | 6.3995E-11 | 5.4465E-11 |
| BP | GO:0000209 | protein polyubiquitination | 94/2594 | 310/18670 | 4.2806E-14 | 6.3995E-11 | 5.4465E-11 |
| BP | GO:0048193 | Golgi vesicle transport | 105/2594 | 368/18670 | 1.1577E-13 | 1.3846E-10 | 1.1784E-10 |
| BP | GO:0070646 | protein modification by small protein removal | 89/2594 | 299/18670 | 6.5382E-13 | 6.5164E-10 | 5.546E-10 |
| BP | GO:0016570 | histone modification | 119/2594 | 454/18670 | 1.6923E-12 | 1.187E-09 | 1.0102E-09 |
| BP | GO:0006914 | autophagy | 127/2594 | 496/18670 | 1.7864E-12 | 1.187E-09 | 1.0102E-09 |
| BP | GO:0061919 | process utilizing autophagic mechanism | 127/2594 | 496/18670 | 1.7864E-12 | 1.187E-09 | 1.0102E-09 |
| BP | GO:0016569 | covalent chromatin modification | 122/2594 | 474/18670 | 3.3753E-12 | 2.0185E-09 | 1.7179E-09 |
| BP | GO:0009896 | positive regulation of catabolic process | 111/2594 | 423/18670 | 8.8425E-12 | 4.8071E-09 | 4.0912E-09 |
| BP | GO:0051169 | nuclear transport | 95/2594 | 346/18670 | 1.8973E-11 | 9.455E-09 | 8.047E-09 |
| BP | GO:0016579 | protein deubiquitination | 82/2594 | 283/18670 | 2.4238E-11 | 1.115E-08 | 9.4892E-09 |
| BP | GO:0051236 | establishment of RNA localization | 63/2594 | 196/18670 | 4.4048E-11 | 1.8815E-08 | 1.6013E-08 |
| BP | GO:0006403 | RNA localization | 70/2594 | 230/18670 | 6.2069E-11 | 2.1663E-08 | 1.8437E-08 |
| BP | GO:0050657 | nucleic acid transport | 62/2594 | 193/18670 | 6.44E-11 | 2.1663E-08 | 1.8437E-08 |
| BP | GO:0050658 | RNA transport | 62/2594 | 193/18670 | 6.44E-11 | 2.1663E-08 | 1.8437E-08 |
| BP | GO:0006913 | nucleocytoplasmic transport | 93/2594 | 343/18670 | 6.5205E-11 | 2.1663E-08 | 1.8437E-08 |
| BP | GO:0031331 | positive regulation of cellular catabolic process | 96/2594 | 361/18670 | 1.032E-10 | 3.248E-08 | 2.7643E-08 |
| BP | GO:0051168 | nuclear export | 61/2594 | 194/18670 | 2.4339E-10 | 7.2775E-08 | 6.1937E-08 |
| BP | GO:0006892 | post-Golgi vesicle-mediated transport | 40/2594 | 104/18670 | 4.1161E-10 | 1.1721E-07 | 9.9757E-08 |
| BP | GO:0016236 | macroautophagy | 81/2594 | 295/18670 | 5.7453E-10 | 1.5617E-07 | 1.3291E-07 |
| BP | GO:0010506 | regulation of autophagy | 87/2594 | 327/18670 | 7.5302E-10 | 1.9579E-07 | 1.6663E-07 |
| BP | GO:0006402 | mRNA catabolic process | 94/2594 | 364/18670 | 8.6493E-10 | 2.1551E-07 | 1.8342E-07 |
| BP | GO:0006401 | RNA catabolic process | 100/2594 | 397/18670 | 1.0882E-09 | 2.6031E-07 | 2.2154E-07 |
| BP | GO:0016197 | endosomal transport | 65/2594 | 224/18670 | 2.5952E-09 | 5.9689E-07 | 5.08E-07 |
| BP | GO:0015931 | nucleobase-containing compound transport | 68/2594 | 241/18670 | 4.0455E-09 | 8.8708E-07 | 7.5498E-07 |
| BP | GO:0000956 | nuclear-transcribed mRNA catabolic process | 61/2594 | 207/18670 | 4.1536E-09 | 8.8708E-07 | 7.5498E-07 |
| BP | GO:0043254 | regulation of protein complex assembly | 111/2594 | 467/18670 | 4.7854E-09 | 9.8396E-07 | 8.3743E-07 |
| BP | GO:0042176 | regulation of protein catabolic process | 95/2594 | 381/18670 | 4.9363E-09 | 9.8396E-07 | 8.3743E-07 |
| BP | GO:0043087 | regulation of GTPase activity | 113/2594 | 479/18670 | 5.4644E-09 | 1.0371E-06 | 8.8269E-07 |
| BP | GO:1903311 | regulation of mRNA metabolic process | 84/2594 | 324/18670 | 5.5499E-09 | 1.0371E-06 | 8.8269E-07 |
| BP | GO:0018205 | peptidyl-lysine modification | 97/2594 | 397/18670 | 1.0341E-08 | 1.8738E-06 | 1.5948E-06 |
| BP | GO:0006611 | protein export from nucleus | 54/2594 | 179/18670 | 1.2992E-08 | 2.2851E-06 | 1.9448E-06 |
| BP | GO:1902115 | regulation of organelle assembly | 57/2594 | 194/18670 | 1.4894E-08 | 2.5448E-06 | 2.1658E-06 |
| BP | GO:0010256 | endomembrane system organization | 104/2594 | 438/18670 | 1.5466E-08 | 2.569E-06 | 2.1865E-06 |
| BP | GO:0051028 | mRNA transport | 48/2594 | 152/18670 | 1.6556E-08 | 2.6758E-06 | 2.2774E-06 |
| BP | GO:0006405 | RNA export from nucleus | 44/2594 | 135/18670 | 2.2571E-08 | 3.5519E-06 | 3.0229E-06 |
| BP | GO:0032984 | protein-containing complex disassembly | 83/2594 | 329/18670 | 2.6029E-08 | 3.9912E-06 | 3.3968E-06 |
| BP | GO:0032508 | DNA duplex unwinding | 38/2594 | 110/18670 | 3.5191E-08 | 5.2611E-06 | 4.4776E-06 |
| BP | GO:0031124 | mRNA 3'-end processing | 35/2594 | 98/18670 | 4.6409E-08 | 6.7689E-06 | 5.7609E-06 |
| BP | GO:0043543 | protein acylation | 66/2594 | 248/18670 | 8.1124E-08 | 1.1283E-05 | 9.6032E-06 |
| BP | GO:0006470 | protein dephosphorylation | 80/2594 | 321/18670 | 8.1135E-08 | 1.1283E-05 | 9.6032E-06 |
| BP | GO:0008380 | RNA splicing | 107/2594 | 469/18670 | 8.8167E-08 | 1.1983E-05 | 1.0198E-05 |
| BP | GO:0042147 | retrograde transport, endosome to Golgi | 32/2594 | 88/18670 | 1.0655E-07 | 1.4159E-05 | 1.2051E-05 |
| BP | GO:0032392 | DNA geometric change | 39/2594 | 119/18670 | 1.1806E-07 | 1.5176E-05 | 1.2916E-05 |
| BP | GO:0007030 | Golgi organization | 44/2594 | 142/18670 | 1.1928E-07 | 1.5176E-05 | 1.2916E-05 |
| BP | GO:0016241 | regulation of macroautophagy | 50/2594 | 171/18670 | 1.3221E-07 | 1.6471E-05 | 1.4018E-05 |
| BP | GO:0000377 | RNA splicing, via transesterification reactions with bulged adenosine as nucleophile | 90/2594 | 379/18670 | 1.4145E-07 | 1.6723E-05 | 1.4232E-05 |
| BP | GO:0000398 | mRNA splicing, via spliceosome | 90/2594 | 379/18670 | 1.4145E-07 | 1.6723E-05 | 1.4232E-05 |
| BP | GO:0070507 | regulation of microtubule cytoskeleton organization | 53/2594 | 186/18670 | 1.4262E-07 | 1.6723E-05 | 1.4232E-05 |
| BP | GO:0018105 | peptidyl-serine phosphorylation | 75/2594 | 299/18670 | 1.5463E-07 | 1.7782E-05 | 1.5134E-05 |
| BP | GO:1903362 | regulation of cellular protein catabolic process | 65/2594 | 247/18670 | 1.5904E-07 | 1.7787E-05 | 1.5138E-05 |
| BP | GO:0033044 | regulation of chromosome organization | 83/2594 | 342/18670 | 1.6062E-07 | 1.7787E-05 | 1.5138E-05 |
| BP | GO:0000375 | RNA splicing, via transesterification reactions | 90/2594 | 382/18670 | 2.0621E-07 | 2.2202E-05 | 1.8896E-05 |
| BP | GO:0046931 | pore complex assembly | 13/2594 | 20/18670 | 2.0791E-07 | 2.2202E-05 | 1.8896E-05 |
| BP | GO:0031503 | protein-containing complex localization | 71/2594 | 281/18670 | 2.4332E-07 | 2.5528E-05 | 2.1726E-05 |
| BP | GO:0006473 | protein acetylation | 56/2594 | 204/18670 | 2.5251E-07 | 2.6034E-05 | 2.2157E-05 |
| BP | GO:0009411 | response to UV | 43/2594 | 141/18670 | 2.7161E-07 | 2.7103E-05 | 2.3067E-05 |
| BP | GO:0071426 | ribonucleoprotein complex export from nucleus | 40/2594 | 127/18670 | 2.7194E-07 | 2.7103E-05 | 2.3067E-05 |
| BP | GO:0051052 | regulation of DNA metabolic process | 98/2594 | 429/18670 | 2.863E-07 | 2.8067E-05 | 2.3887E-05 |
| BP | GO:0030705 | cytoskeleton-dependent intracellular transport | 51/2594 | 180/18670 | 2.9547E-07 | 2.8499E-05 | 2.4255E-05 |
| BP | GO:0009144 | purine nucleoside triphosphate metabolic process | 82/2594 | 342/18670 | 3.277E-07 | 3.1105E-05 | 2.6473E-05 |
| BP | GO:0007034 | vacuolar transport | 43/2594 | 142/18670 | 3.3777E-07 | 3.1557E-05 | 2.6858E-05 |
| BP | GO:0071166 | ribonucleoprotein complex localization | 40/2594 | 128/18670 | 3.4301E-07 | 3.1557E-05 | 2.6858E-05 |
| BP | GO:0018209 | peptidyl-serine modification | 78/2594 | 322/18670 | 4.0687E-07 | 3.6521E-05 | 3.1082E-05 |
| BP | GO:0006378 | mRNA polyadenylation | 20/2594 | 44/18670 | 4.0918E-07 | 3.6521E-05 | 3.1082E-05 |
| BP | GO:0043547 | positive regulation of GTPase activity | 93/2594 | 405/18670 | 4.4763E-07 | 3.9365E-05 | 3.3503E-05 |
| BP | GO:0009167 | purine ribonucleoside monophosphate metabolic process | 81/2594 | 340/18670 | 5.1042E-07 | 4.4237E-05 | 3.7649E-05 |
| BP | GO:0009205 | purine ribonucleoside triphosphate metabolic process | 80/2594 | 335/18670 | 5.4026E-07 | 4.6154E-05 | 3.9281E-05 |
| BP | GO:0009126 | purine nucleoside monophosphate metabolic process | 81/2594 | 341/18670 | 5.7983E-07 | 4.8814E-05 | 4.1544E-05 |
| BP | GO:1903050 | regulation of proteolysis involved in cellular protein catabolic process | 57/2594 | 214/18670 | 5.8772E-07 | 4.8814E-05 | 4.1544E-05 |
| BP | GO:0016050 | vesicle organization | 78/2594 | 325/18670 | 6.0409E-07 | 4.9485E-05 | 4.2116E-05 |
| BP | GO:0010508 | positive regulation of autophagy | 37/2594 | 117/18670 | 6.7209E-07 | 5.4312E-05 | 4.6224E-05 |
| BP | GO:0072665 | protein localization to vacuole | 25/2594 | 65/18670 | 7.6422E-07 | 6.0934E-05 | 5.186E-05 |
| BP | GO:0016311 | dephosphorylation | 105/2594 | 478/18670 | 8.0915E-07 | 6.3667E-05 | 5.4186E-05 |
| BP | GO:0008334 | histone mRNA metabolic process | 14/2594 | 25/18670 | 9.4968E-07 | 7.321E-05 | 6.2308E-05 |
| BP | GO:0031330 | negative regulation of cellular catabolic process | 66/2594 | 264/18670 | 9.5491E-07 | 7.321E-05 | 6.2308E-05 |
| BP | GO:0043631 | RNA polyadenylation | 20/2594 | 46/18670 | 9.856E-07 | 7.4606E-05 | 6.3496E-05 |
| BP | GO:0009141 | nucleoside triphosphate metabolic process | 84/2594 | 362/18670 | 1.0133E-06 | 7.5746E-05 | 6.4466E-05 |
| BP | GO:0006352 | DNA-templated transcription, initiation | 63/2594 | 249/18670 | 1.0788E-06 | 7.9642E-05 | 6.7782E-05 |
| BP | GO:0009199 | ribonucleoside triphosphate metabolic process | 80/2594 | 341/18670 | 1.1503E-06 | 8.3889E-05 | 7.1396E-05 |
| BP | GO:0061136 | regulation of proteasomal protein catabolic process | 50/2594 | 183/18670 | 1.2622E-06 | 9.0366E-05 | 7.6909E-05 |
| BP | GO:0009123 | nucleoside monophosphate metabolic process | 86/2594 | 375/18670 | 1.2693E-06 | 9.0366E-05 | 7.6909E-05 |
| BP | GO:0061013 | regulation of mRNA catabolic process | 53/2594 | 199/18670 | 1.4443E-06 | 0.00010161 | 8.6482E-05 |
| BP | GO:0009161 | ribonucleoside monophosphate metabolic process | 82/2594 | 354/18670 | 1.4614E-06 | 0.00010162 | 8.6488E-05 |
| BP | GO:0006997 | nucleus organization | 39/2594 | 130/18670 | 1.5137E-06 | 0.00010404 | 8.855E-05 |
| BP | GO:0048524 | positive regulation of viral process | 34/2594 | 107/18670 | 1.6618E-06 | 0.00011194 | 9.5272E-05 |
| BP | GO:0031334 | positive regulation of protein complex assembly | 66/2594 | 268/18670 | 1.6794E-06 | 0.00011194 | 9.5272E-05 |
| BP | GO:1903320 | regulation of protein modification by small protein conjugation or removal | 59/2594 | 231/18670 | 1.6847E-06 | 0.00011194 | 9.5272E-05 |
| BP | GO:0031123 | RNA 3'-end processing | 43/2594 | 150/18670 | 1.7408E-06 | 0.00011439 | 9.7357E-05 |
| BP | GO:0071826 | ribonucleoprotein complex subunit organization | 70/2594 | 291/18670 | 2.0511E-06 | 0.00013332 | 0.00011347 |
| BP | GO:0010970 | transport along microtubule | 45/2594 | 161/18670 | 2.1601E-06 | 0.00013742 | 0.00011695 |
| BP | GO:0099111 | microtubule-based transport | 45/2594 | 161/18670 | 2.1601E-06 | 0.00013742 | 0.00011695 |
| BP | GO:1902903 | regulation of supramolecular fiber organization | 81/2594 | 352/18670 | 2.2232E-06 | 0.00013957 | 0.00011878 |
| BP | GO:0009895 | negative regulation of catabolic process | 73/2594 | 308/18670 | 2.2405E-06 | 0.00013957 | 0.00011878 |
| BP | GO:0006623 | protein targeting to vacuole | 17/2594 | 37/18670 | 2.5187E-06 | 0.00015528 | 0.00013216 |
| BP | GO:0022618 | ribonucleoprotein complex assembly | 67/2594 | 277/18670 | 2.7492E-06 | 0.00016776 | 0.00014278 |
| BP | GO:0031023 | microtubule organizing center organization | 39/2594 | 133/18670 | 2.8249E-06 | 0.00017064 | 0.00014523 |
| BP | GO:0046034 | ATP metabolic process | 72/2594 | 305/18670 | 3.0563E-06 | 0.00018277 | 0.00015555 |
| BP | GO:2000058 | regulation of ubiquitin-dependent protein catabolic process | 42/2594 | 149/18670 | 3.6729E-06 | 0.00021746 | 0.00018508 |
| BP | GO:0007041 | lysosomal transport | 33/2594 | 106/18670 | 3.8818E-06 | 0.00022758 | 0.00019369 |
| BP | GO:0006513 | protein monoubiquitination | 24/2594 | 66/18670 | 4.0544E-06 | 0.0002335 | 0.00019873 |
| BP | GO:0016072 | rRNA metabolic process | 62/2594 | 253/18670 | 4.0609E-06 | 0.0002335 | 0.00019873 |
| BP | GO:0006119 | oxidative phosphorylation | 41/2594 | 145/18670 | 4.3801E-06 | 0.00024935 | 0.00021222 |
| BP | GO:0042254 | ribosome biogenesis | 70/2594 | 297/18670 | 4.4199E-06 | 0.00024935 | 0.00021222 |
| BP | GO:0018107 | peptidyl-threonine phosphorylation | 37/2594 | 126/18670 | 4.8464E-06 | 0.00027086 | 0.00023052 |
| BP | GO:0043414 | macromolecule methylation | 72/2594 | 309/18670 | 5.0047E-06 | 0.00027711 | 0.00023585 |
| BP | GO:0000910 | cytokinesis | 46/2594 | 171/18670 | 5.2059E-06 | 0.00028561 | 0.00024307 |
| BP | GO:0032886 | regulation of microtubule-based process | 55/2594 | 218/18670 | 5.5487E-06 | 0.00030165 | 0.00025673 |
| BP | GO:0031440 | regulation of mRNA 3'-end processing | 14/2594 | 28/18670 | 5.6667E-06 | 0.00030529 | 0.00025982 |
| BP | GO:0032434 | regulation of proteasomal ubiquitin-dependent protein catabolic process | 36/2594 | 122/18670 | 5.73E-06 | 0.00030594 | 0.00026038 |
| BP | GO:0051258 | protein polymerization | 67/2594 | 283/18670 | 5.9823E-06 | 0.00031658 | 0.00026944 |
| BP | GO:0050684 | regulation of mRNA processing | 39/2594 | 137/18670 | 6.2196E-06 | 0.00032626 | 0.00027767 |
| BP | GO:0019827 | stem cell population maintenance | 43/2594 | 157/18670 | 6.3573E-06 | 0.00033058 | 0.00028135 |
| BP | GO:0034470 | ncRNA processing | 85/2594 | 384/18670 | 6.4525E-06 | 0.00033264 | 0.0002831 |
| BP | GO:0019058 | viral life cycle | 75/2594 | 328/18670 | 6.629E-06 | 0.00033882 | 0.00028836 |
| BP | GO:0006367 | transcription initiation from RNA polymerase II promoter | 49/2594 | 188/18670 | 6.7937E-06 | 0.00034429 | 0.00029302 |
| BP | GO:0043487 | regulation of RNA stability | 48/2594 | 183/18670 | 6.9713E-06 | 0.00035032 | 0.00029815 |
| BP | GO:0048813 | dendrite morphogenesis | 40/2594 | 143/18670 | 7.5933E-06 | 0.0003784 | 0.00032205 |
| BP | GO:0000288 | nuclear-transcribed mRNA catabolic process, deadenylation-dependent decay | 26/2594 | 77/18670 | 7.8954E-06 | 0.0003902 | 0.00033209 |
| BP | GO:0090305 | nucleic acid phosphodiester bond hydrolysis | 70/2594 | 302/18670 | 8.147E-06 | 0.00039934 | 0.00033987 |
| BP | GO:2001252 | positive regulation of chromosome organization | 46/2594 | 174/18670 | 8.5802E-06 | 0.00041715 | 0.00035503 |
| BP | GO:0018394 | peptidyl-lysine acetylation | 45/2594 | 169/18670 | 8.7496E-06 | 0.00042196 | 0.00035912 |
| BP | GO:0018210 | peptidyl-threonine modification | 38/2594 | 134/18670 | 8.9682E-06 | 0.00042696 | 0.00036338 |
| BP | GO:0098727 | maintenance of cell number | 43/2594 | 159/18670 | 9.0057E-06 | 0.00042696 | 0.00036338 |
| BP | GO:0016925 | protein sumoylation | 27/2594 | 82/18670 | 9.0676E-06 | 0.00042696 | 0.00036338 |
| BP | GO:0072666 | establishment of protein localization to vacuole | 19/2594 | 48/18670 | 9.7853E-06 | 0.00045715 | 0.00038908 |
| BP | GO:0016358 | dendrite development | 57/2594 | 233/18670 | 1.031E-05 | 0.00047488 | 0.00040416 |
| BP | GO:0043624 | cellular protein complex disassembly | 54/2594 | 217/18670 | 1.0323E-05 | 0.00047488 | 0.00040416 |
| BP | GO:0006888 | ER to Golgi vesicle-mediated transport | 53/2594 | 212/18670 | 1.0782E-05 | 0.00049219 | 0.00041889 |
| BP | GO:0043928 | exonucleolytic nuclear-transcribed mRNA catabolic process involved in deadenylation-dependent decay | 15/2594 | 33/18670 | 1.1581E-05 | 0.00051826 | 0.00044108 |
| BP | GO:0006406 | mRNA export from nucleus | 33/2594 | 111/18670 | 1.1613E-05 | 0.00051826 | 0.00044108 |
| BP | GO:0071427 | mRNA-containing ribonucleoprotein complex export from nucleus | 33/2594 | 111/18670 | 1.1613E-05 | 0.00051826 | 0.00044108 |
| BP | GO:1903902 | positive regulation of viral life cycle | 22/2594 | 61/18670 | 1.1791E-05 | 0.00052232 | 0.00044453 |
| BP | GO:0007044 | cell-substrate junction assembly | 30/2594 | 97/18670 | 1.2149E-05 | 0.00053032 | 0.00045134 |
| BP | GO:0042775 | mitochondrial ATP synthesis coupled electron transport | 30/2594 | 97/18670 | 1.2149E-05 | 0.00053032 | 0.00045134 |
| BP | GO:0098927 | vesicle-mediated transport between endosomal compartments | 17/2594 | 41/18670 | 1.3809E-05 | 0.00059738 | 0.00050842 |
| BP | GO:0043488 | regulation of mRNA stability | 46/2594 | 177/18670 | 1.3886E-05 | 0.00059738 | 0.00050842 |
| BP | GO:0016574 | histone ubiquitination | 18/2594 | 45/18670 | 1.408E-05 | 0.0006014 | 0.00051184 |
| BP | GO:0006368 | transcription elongation from RNA polymerase II promoter | 27/2594 | 84/18670 | 1.495E-05 | 0.00063147 | 0.00053743 |
| BP | GO:0006353 | DNA-templated transcription, termination | 25/2594 | 75/18670 | 1.5134E-05 | 0.00063147 | 0.00053743 |
| BP | GO:0043902 | positive regulation of multi-organism process | 48/2594 | 188/18670 | 1.5201E-05 | 0.00063147 | 0.00053743 |
| BP | GO:0042773 | ATP synthesis coupled electron transport | 30/2594 | 98/18670 | 1.5206E-05 | 0.00063147 | 0.00053743 |
| BP | GO:0031396 | regulation of protein ubiquitination | 51/2594 | 204/18670 | 1.5666E-05 | 0.0006461 | 0.00054988 |
| BP | GO:0043244 | regulation of protein complex disassembly | 33/2594 | 113/18670 | 1.7527E-05 | 0.00071787 | 0.00061097 |
| BP | GO:0045022 | early endosome to late endosome transport | 16/2594 | 38/18670 | 1.9567E-05 | 0.00079409 | 0.00067584 |
| BP | GO:0006999 | nuclear pore organization | 9/2594 | 14/18670 | 1.9653E-05 | 0.00079409 | 0.00067584 |
| BP | GO:0010639 | negative regulation of organelle organization | 85/2594 | 395/18670 | 1.9868E-05 | 0.00079511 | 0.00067671 |
| BP | GO:0046822 | regulation of nucleocytoplasmic transport | 31/2594 | 104/18670 | 1.9944E-05 | 0.00079511 | 0.00067671 |
| BP | GO:1900363 | regulation of mRNA polyadenylation | 10/2594 | 17/18670 | 2.0099E-05 | 0.00079596 | 0.00067743 |
| BP | GO:0034333 | adherens junction assembly | 28/2594 | 90/18670 | 2.0732E-05 | 0.00081564 | 0.00069418 |
| BP | GO:0006893 | Golgi to plasma membrane transport | 22/2594 | 63/18670 | 2.1243E-05 | 0.0008303 | 0.00070665 |
| BP | GO:0007045 | cell-substrate adherens junction assembly | 26/2594 | 81/18670 | 2.1962E-05 | 0.0008413 | 0.00071602 |
| BP | GO:0032204 | regulation of telomere maintenance | 26/2594 | 81/18670 | 2.1962E-05 | 0.0008413 | 0.00071602 |
| BP | GO:0048041 | focal adhesion assembly | 26/2594 | 81/18670 | 2.1962E-05 | 0.0008413 | 0.00071602 |
| BP | GO:0031146 | SCF-dependent proteasomal ubiquitin-dependent protein catabolic process | 29/2594 | 95/18670 | 2.2227E-05 | 0.0008413 | 0.00071602 |
| BP | GO:0007098 | centrosome cycle | 35/2594 | 124/18670 | 2.2228E-05 | 0.0008413 | 0.00071602 |
| CC | GO:0000151 | ubiquitin ligase complex | 86/2673 | 282/19717 | 9.2978E-14 | 6.8525E-11 | 4.8251E-11 |
| CC | GO:0005635 | nuclear envelope | 119/2673 | 464/19717 | 1.6062E-12 | 5.9187E-10 | 4.1676E-10 |
| CC | GO:0031965 | nuclear membrane | 82/2673 | 296/19717 | 8.4554E-11 | 2.0772E-08 | 1.4626E-08 |
| CC | GO:0016607 | nuclear speck | 100/2673 | 397/19717 | 2.8768E-10 | 5.3004E-08 | 3.7322E-08 |
| CC | GO:0061695 | transferase complex, transferring phosphorus-containing groups | 71/2673 | 259/19717 | 2.5074E-09 | 3.6959E-07 | 2.6024E-07 |
| CC | GO:0031252 | cell leading edge | 97/2673 | 403/19717 | 6.7654E-09 | 8.3102E-07 | 5.8515E-07 |
| CC | GO:0031461 | cullin-RING ubiquitin ligase complex | 49/2673 | 158/19717 | 1.0256E-08 | 1.0799E-06 | 7.6036E-07 |
| CC | GO:0098687 | chromosomal region | 84/2673 | 349/19717 | 6.8556E-08 | 6.1169E-06 | 4.3071E-06 |
| CC | GO:0005925 | focal adhesion | 94/2673 | 405/19717 | 7.4697E-08 | 6.1169E-06 | 4.3071E-06 |
| CC | GO:0030055 | cell-substrate junction | 95/2673 | 412/19717 | 8.8679E-08 | 6.3023E-06 | 4.4376E-06 |
| CC | GO:0030027 | lamellipodium | 54/2673 | 193/19717 | 9.4064E-08 | 6.3023E-06 | 4.4376E-06 |
| CC | GO:0005924 | cell-substrate adherens junction | 94/2673 | 408/19717 | 1.0786E-07 | 6.6241E-06 | 4.6643E-06 |
| CC | GO:0034708 | methyltransferase complex | 37/2673 | 113/19717 | 1.3563E-07 | 7.1784E-06 | 5.0546E-06 |
| CC | GO:0005802 | trans-Golgi network | 62/2673 | 236/19717 | 1.3636E-07 | 7.1784E-06 | 5.0546E-06 |
| CC | GO:0005819 | spindle | 82/2673 | 347/19717 | 2.249E-07 | 1.0959E-05 | 7.7163E-06 |
| CC | GO:0000775 | chromosome, centromeric region | 53/2673 | 193/19717 | 2.3791E-07 | 1.0959E-05 | 7.7163E-06 |
| CC | GO:0031248 | protein acetyltransferase complex | 32/2673 | 95/19717 | 4.5424E-07 | 1.8599E-05 | 1.3096E-05 |
| CC | GO:1902493 | acetyltransferase complex | 32/2673 | 95/19717 | 4.5424E-07 | 1.8599E-05 | 1.3096E-05 |
| CC | GO:0035770 | ribonucleoprotein granule | 57/2673 | 223/19717 | 1.1236E-06 | 4.3584E-05 | 3.0689E-05 |
| CC | GO:0005938 | cell cortex | 72/2673 | 308/19717 | 1.8357E-06 | 6.7645E-05 | 4.7631E-05 |
| CC | GO:0005681 | spliceosomal complex | 49/2673 | 185/19717 | 2.0917E-06 | 7.3408E-05 | 5.1689E-05 |
| CC | GO:0036464 | cytoplasmic ribonucleoprotein granule | 54/2673 | 212/19717 | 2.383E-06 | 7.9832E-05 | 5.6212E-05 |
| CC | GO:0005769 | early endosome | 79/2673 | 350/19717 | 2.5307E-06 | 8.1093E-05 | 5.71E-05 |
| CC | GO:0120111 | neuron projection cytoplasm | 28/2673 | 84/19717 | 2.946E-06 | 8.7305E-05 | 6.1474E-05 |
| CC | GO:0098798 | mitochondrial protein complex | 63/2673 | 262/19717 | 2.9615E-06 | 8.7305E-05 | 6.1474E-05 |
| CC | GO:0001726 | ruffle | 46/2673 | 172/19717 | 3.1876E-06 | 9.0355E-05 | 6.3622E-05 |
| CC | GO:0030496 | midbody | 46/2673 | 173/19717 | 3.7788E-06 | 0.00010315 | 7.263E-05 |
| CC | GO:0016605 | PML body | 31/2673 | 99/19717 | 3.9646E-06 | 0.00010435 | 7.3478E-05 |
| CC | GO:0080008 | Cul4-RING E3 ubiquitin ligase complex | 15/2673 | 33/19717 | 8.5476E-06 | 0.00021202 | 0.00014929 |
| CC | GO:1904115 | axon cytoplasm | 21/2673 | 57/19717 | 8.6305E-06 | 0.00021202 | 0.00014929 |
| CC | GO:0010494 | cytoplasmic stress granule | 23/2673 | 66/19717 | 9.6121E-06 | 0.00022138 | 0.00015588 |
| CC | GO:0099023 | tethering complex | 23/2673 | 66/19717 | 9.6121E-06 | 0.00022138 | 0.00015588 |
| CC | GO:0005643 | nuclear pore | 28/2673 | 89/19717 | 1.0347E-05 | 0.00023109 | 0.00016272 |
| CC | GO:0000123 | histone acetyltransferase complex | 27/2673 | 85/19717 | 1.2141E-05 | 0.00026316 | 0.0001853 |
| CC | GO:1902911 | protein kinase complex | 32/2673 | 109/19717 | 1.2579E-05 | 0.00026487 | 0.0001865 |
| CC | GO:0035097 | histone methyltransferase complex | 27/2673 | 86/19717 | 1.5431E-05 | 0.00031591 | 0.00022245 |
| CC | GO:0019005 | SCF ubiquitin ligase complex | 22/2673 | 64/19717 | 1.9119E-05 | 0.00036171 | 0.00025469 |
| CC | GO:0018995 | host | 24/2673 | 73/19717 | 1.9141E-05 | 0.00036171 | 0.00025469 |
| CC | GO:0043657 | host cell | 24/2673 | 73/19717 | 1.9141E-05 | 0.00036171 | 0.00025469 |
| CC | GO:0000922 | spindle pole | 42/2673 | 164/19717 | 2.6159E-05 | 0.00048198 | 0.00033938 |
| CC | GO:0030426 | growth cone | 43/2673 | 171/19717 | 3.3901E-05 | 0.00060939 | 0.00042909 |
| CC | GO:0000776 | kinetochore | 36/2673 | 135/19717 | 3.8283E-05 | 0.00065629 | 0.00046212 |
| CC | GO:0005684 | U2-type spliceosomal complex | 27/2673 | 90/19717 | 3.8291E-05 | 0.00065629 | 0.00046212 |
| CC | GO:0070603 | SWI/SNF superfamily-type complex | 24/2673 | 76/19717 | 4.0455E-05 | 0.00067763 | 0.00047714 |
| CC | GO:0030117 | membrane coat | 28/2673 | 96/19717 | 4.8755E-05 | 0.00077149 | 0.00054323 |
| CC | GO:0048475 | coated membrane | 28/2673 | 96/19717 | 4.8755E-05 | 0.00077149 | 0.00054323 |
| CC | GO:0044215 | other organism | 24/2673 | 77/19717 | 5.1293E-05 | 0.00077149 | 0.00054323 |
| CC | GO:0044216 | other organism cell | 24/2673 | 77/19717 | 5.1293E-05 | 0.00077149 | 0.00054323 |
| CC | GO:0044217 | other organism part | 24/2673 | 77/19717 | 5.1293E-05 | 0.00077149 | 0.00054323 |
| CC | GO:0071013 | catalytic step 2 spliceosome | 26/2673 | 87/19717 | 5.6858E-05 | 0.00083809 | 0.00059013 |
| CC | GO:0071007 | U2-type catalytic step 2 spliceosome | 13/2673 | 30/19717 | 6.352E-05 | 0.00091792 | 0.00064634 |
| CC | GO:0030427 | site of polarized growth | 43/2673 | 176/19717 | 7.0165E-05 | 0.00099445 | 0.00070023 |
| MF | GO:0004842 | ubiquitin-protein transferase activity | 122/2603 | 382/17697 | 6.3199E-18 | 5.6896E-15 | 4.7935E-15 |
| MF | GO:0019787 | ubiquitin-like protein transferase activity | 127/2603 | 407/17697 | 1.0817E-17 | 5.6896E-15 | 4.7935E-15 |
| MF | GO:0017016 | Ras GTPase binding | 117/2603 | 429/17697 | 6.6497E-12 | 2.3318E-09 | 1.9646E-09 |
| MF | GO:0031267 | small GTPase binding | 119/2603 | 443/17697 | 1.2934E-11 | 3.4016E-09 | 2.8658E-09 |
| MF | GO:0004386 | helicase activity | 58/2603 | 163/17697 | 2.5411E-11 | 5.3465E-09 | 4.5044E-09 |
| MF | GO:0043021 | ribonucleoprotein complex binding | 50/2603 | 133/17697 | 6.1378E-11 | 1.0762E-08 | 9.0667E-09 |
| MF | GO:0061659 | ubiquitin-like protein ligase activity | 71/2603 | 230/17697 | 2.9587E-10 | 4.3242E-08 | 3.6432E-08 |
| MF | GO:0004674 | protein serine/threonine kinase activity | 114/2603 | 439/17697 | 3.2884E-10 | 4.3242E-08 | 3.6432E-08 |
| MF | GO:0042393 | histone binding | 63/2603 | 197/17697 | 6.0228E-10 | 7.0399E-08 | 5.9312E-08 |
| MF | GO:0061630 | ubiquitin protein ligase activity | 68/2603 | 221/17697 | 8.1555E-10 | 8.5796E-08 | 7.2283E-08 |
| MF | GO:0140030 | modification-dependent protein binding | 50/2603 | 144/17697 | 1.553E-09 | 1.4852E-07 | 1.2513E-07 |
| MF | GO:0034212 | peptide N-acetyltransferase activity | 29/2603 | 75/17697 | 3.0798E-07 | 2.7E-05 | 2.2748E-05 |
| MF | GO:0045296 | cadherin binding | 83/2603 | 331/17697 | 4.1345E-07 | 3.3458E-05 | 2.8188E-05 |
| MF | GO:0019783 | ubiquitin-like protein-specific protease activity | 40/2603 | 123/17697 | 4.7925E-07 | 3.6012E-05 | 3.0341E-05 |
| MF | GO:0003713 | transcription coactivator activity | 80/2603 | 319/17697 | 6.62E-07 | 4.6189E-05 | 3.8914E-05 |
| MF | GO:0030695 | GTPase regulator activity | 77/2603 | 304/17697 | 7.0249E-07 | 4.6189E-05 | 3.8914E-05 |
| MF | GO:0060589 | nucleoside-triphosphatase regulator activity | 84/2603 | 344/17697 | 1.1481E-06 | 7.1044E-05 | 5.9855E-05 |
| MF | GO:0005085 | guanyl-nucleotide exchange factor activity | 58/2603 | 214/17697 | 1.6531E-06 | 9.6617E-05 | 8.14E-05 |
| MF | GO:0140098 | catalytic activity, acting on RNA | 91/2603 | 386/17697 | 2.0927E-06 | 0.00011587 | 9.7621E-05 |
| MF | GO:0003724 | RNA helicase activity | 28/2603 | 78/17697 | 2.8022E-06 | 0.00014739 | 0.00012418 |
| MF | GO:0061733 | peptide-lysine-N-acetyltransferase activity | 25/2603 | 66/17697 | 3.0868E-06 | 0.00015463 | 0.00013028 |
| MF | GO:0032182 | ubiquitin-like protein binding | 32/2603 | 96/17697 | 3.5862E-06 | 0.00017149 | 0.00014448 |
| MF | GO:0005088 | Ras guanyl-nucleotide exchange factor activity | 41/2603 | 137/17697 | 3.8581E-06 | 0.00017502 | 0.00014746 |
| MF | GO:0031593 | polyubiquitin modification-dependent protein binding | 21/2603 | 51/17697 | 3.9929E-06 | 0.00017502 | 0.00014746 |
| MF | GO:0005096 | GTPase activator activity | 68/2603 | 273/17697 | 5.6918E-06 | 0.00023951 | 0.00020179 |
| MF | GO:0004402 | histone acetyltransferase activity | 24/2603 | 64/17697 | 5.9356E-06 | 0.00024017 | 0.00020234 |
| MF | GO:0008080 | N-acetyltransferase activity | 30/2603 | 90/17697 | 7.15E-06 | 0.00027858 | 0.00023471 |
| MF | GO:0008094 | DNA-dependent ATPase activity | 25/2603 | 69/17697 | 7.8011E-06 | 0.00029085 | 0.00024504 |
| MF | GO:0070530 | K63-linked polyubiquitin modification-dependent protein binding | 12/2603 | 21/17697 | 8.0177E-06 | 0.00029085 | 0.00024504 |
| MF | GO:0035091 | phosphatidylinositol binding | 62/2603 | 245/17697 | 8.4709E-06 | 0.00029704 | 0.00025026 |
| MF | GO:0016407 | acetyltransferase activity | 34/2603 | 110/17697 | 1.172E-05 | 0.00039772 | 0.00033508 |
| MF | GO:0043130 | ubiquitin binding | 26/2603 | 76/17697 | 1.6992E-05 | 0.00055862 | 0.00047064 |
| MF | GO:0017137 | Rab GTPase binding | 47/2603 | 175/17697 | 1.9907E-05 | 0.00063461 | 0.00053466 |
| MF | GO:0036459 | thiol-dependent ubiquitinyl hydrolase activity | 34/2603 | 114/17697 | 2.7086E-05 | 0.00081412 | 0.0006859 |
| MF | GO:0101005 | ubiquitinyl hydrolase activity | 34/2603 | 114/17697 | 2.7086E-05 | 0.00081412 | 0.0006859 |
