# Supplementary material for: PIK3C2A is a prognostic biomarker that is linked to immune infiltrates in kidney renal clear cell carcinoma
Source: Front Immunol. 2023 Mar 30;14:1114572. doi: 10.3389/fimmu.2023.1114572 (PMC10098324; doi:10.3389/fimmu.2023.1114572)
Supplement: Supplementary file 3 [file Table_3.docx]

| **Supplementary table 3** The results of KEGG enrichment analysis of the PCGs co-expressed with PIK3C2A. | | | | | | | | |
| --- | --- | --- | --- | --- | --- | --- | --- | --- |
| ONTOLOGY |  | ID | Description | GeneRatio | BgRatio | pvalue | p.adjust | qvalue |
| KEGG |  | hsa05168 | Herpes simplex virus 1 infection | 143/1171 | 498/8076 | 1.7881E-17 | 5.4E-15 | 3.8773E-15 |
| KEGG |  | hsa04120 | Ubiquitin mediated proteolysis | 48/1171 | 140/8076 | 2.6463E-09 | 3.9959E-07 | 2.8691E-07 |
| KEGG |  | hsa05211 | Renal cell carcinoma | 28/1171 | 69/8076 | 1.0031E-07 | 1.0098E-05 | 7.2507E-06 |
| KEGG |  | hsa05010 | Alzheimer disease | 90/1171 | 369/8076 | 1.8128E-07 | 1.3687E-05 | 9.8273E-06 |
| KEGG |  | hsa01521 | EGFR tyrosine kinase inhibitor resistance | 29/1171 | 79/8076 | 7.5012E-07 | 4.5307E-05 | 3.2532E-05 |
| KEGG |  | hsa04714 | Thermogenesis | 61/1171 | 231/8076 | 1.1487E-06 | 5.7816E-05 | 4.1513E-05 |
| KEGG |  | hsa05210 | Colorectal cancer | 30/1171 | 86/8076 | 1.6943E-06 | 7.3095E-05 | 5.2484E-05 |
| KEGG |  | hsa05016 | Huntington disease | 74/1171 | 306/8076 | 3.2501E-06 | 0.00012269 | 8.8095E-05 |
| KEGG |  | hsa04071 | Sphingolipid signaling pathway | 36/1171 | 119/8076 | 7.3743E-06 | 0.0002227 | 0.00015991 |
| KEGG |  | hsa04722 | Neurotrophin signaling pathway | 36/1171 | 119/8076 | 7.3743E-06 | 0.0002227 | 0.00015991 |
| KEGG |  | hsa05014 | Amyotrophic lateral sclerosis | 83/1171 | 364/8076 | 9.6946E-06 | 0.00026616 | 0.00019111 |
| KEGG |  | hsa04068 | FoxO signaling pathway | 38/1171 | 131/8076 | 1.232E-05 | 0.00028701 | 0.00020608 |
| KEGG |  | hsa05220 | Chronic myeloid leukemia | 26/1171 | 76/8076 | 1.2355E-05 | 0.00028701 | 0.00020608 |
| KEGG |  | hsa04140 | Autophagy - animal | 39/1171 | 137/8076 | 1.5427E-05 | 0.0003106 | 0.00022302 |
| KEGG |  | hsa04910 | Insulin signaling pathway | 39/1171 | 137/8076 | 1.5427E-05 | 0.0003106 | 0.00022302 |
| KEGG |  | hsa05215 | Prostate cancer | 30/1171 | 97/8076 | 2.6167E-05 | 0.00048689 | 0.00034959 |
| KEGG |  | hsa04150 | mTOR signaling pathway | 42/1171 | 155/8076 | 2.7407E-05 | 0.00048689 | 0.00034959 |
| KEGG |  | hsa05135 | Yersinia infection | 38/1171 | 137/8076 | 3.7392E-05 | 0.000603 | 0.00043297 |
| KEGG |  | hsa05161 | Hepatitis B | 43/1171 | 162/8076 | 3.7937E-05 | 0.000603 | 0.00043297 |
| KEGG |  | hsa04144 | Endocytosis | 60/1171 | 252/8076 | 4.5269E-05 | 0.00068357 | 0.00049081 |
| KEGG |  | hsa04935 | Growth hormone synthesis, secretion and action | 34/1171 | 119/8076 | 4.9181E-05 | 0.00070726 | 0.00050783 |
| KEGG |  | hsa04919 | Thyroid hormone signaling pathway | 34/1171 | 121/8076 | 7.1186E-05 | 0.00097719 | 0.00070164 |
